# Breast Cancer Cell Invasion into a Three Dimensional Tumor-Stroma Microenvironment

**DOI:** 10.1038/srep34094

**Published:** 2016-09-28

**Authors:** Danh Truong, Julieann Puleo, Alison Llave, Ghassan Mouneimne, Roger D. Kamm, Mehdi Nikkhah

**Affiliations:** 1 School of Biological and Health Systems Engineering (SBHSE), Arizona State University, Tempe, Arizona 85287, USA; 2 University of Arizona Cancer Center, Department of Cellular and Molecular Medicine, Tucson, Arizona 85724, USA; 3 Department of Biological Engineering, Massachusetts Institute of Technology, Cambridge, MA, USA; 4 Mechanical Engineering, Massachusetts Institute of Technology, Cambridge, MA, USA.

## Abstract

In this study, to model 3D chemotactic tumor-stroma invasion *in vitro*, we developed an innovative microfluidic chip allowing side-by-side positioning of 3D hydrogel-based matrices. We were able to (1) create a dual matrix architecture that extended in a continuous manner, thus allowing invasion from one 3D matrix to another, and (2) establish distinct regions of tumor and stroma cell/ECM compositions, with a clearly demarcated tumor invasion front, thus allowing us to quantitatively analyze progression of cancer cells into the stroma at a tissue or single-cell level. We showed significantly enhanced cancer cell invasion in response to a transient gradient of epidermal growth factor (EGF). 3D tracking at the single-cell level displayed increased migration speed and persistence. Subsequently, we analyzed changes in expression of EGF receptors, cell aspect ratio, and protrusive activity. These findings show the unique ability of our model to quantitatively analyze 3D chemotactic invasion, both globally by tracking the progression of the invasion front, and at the single-cell level by examining changes in cellular behavior and morphology using high-resolution imaging. Taken together, we have shown a novel model recapitulating 3D tumor-stroma interactions for studies of real-time cell invasion and morphological changes within a single platform.

Breast cancer is the one of leading causes of cancer-related death among women in the United States^1^. This disease progresses in many steps ranging from tumor growth, stroma invasion, and spreading throughout the body^2^,^3^. Invasion into the surrounding stroma begins a process of interactions between tumor cells and the stroma by cellular crosstalk and paracrine signaling (i.e. fibroblasts, pericytes, immune cells, endothelial cells, etc.) and is influenced by biochemical/biophysical cues (i.e. drug and nutrient transport, ECM composition and stiffness, etc.) within the stroma^2^,^4^,^5^,^6^. Despite significant advances in therapeutic regimens, anti-cancer drugs often fail due to lack of comprehensive preclinical studies utilizing models incorporating the complexities of the native tumor-stroma microenvironment^7^,^8^,^9^,^10^,^11^. In this regard, the interactions that specifically arise from a variety of biochemical and biophysical gradients, and cellular components should not be overlooked when developing *in vitro* tumor microenvironment models^12^.

Chemoattractants, such as epidermal growth factor (EGF), are aggressive drivers of cancer invasion by activating cell membrane receptors and intracellular pathways that provide guidance and motility cues to the cells^3^. For example, cancer cells have been shown to secrete colony stimulating factor-1 (CSF-1), which then causes macrophages to produce gradients of EGF^13^,^14^. This would often lead to enhanced proliferation, survival, and motility of cancer cells^15^,^16^,^17^,^18^. For example, micro-needles filled with Matrigel^®^ and EGF inserted into the mouse fat pads attracted breast cancer cells to the site of injection. However, this model required expensive imaging, such as multiphoton laser-scanning and second harmonic generation^19^,^20^, to observe the effect of EGF on cancer invasion in real-time^18^. Furthermore, animal models do not allow decoupled control of cell-cell and cell-ECM interactions creating significant difficulties in elucidating the role of each separate stromal component. For instance, cancer cells *in vivo* have been shown to migrate toward one specific areas of vascularization. However, it was unclear whether the cancer cell’s response was due to the sole role of biochemical (i.e. chemoattractants) or biophysical (i.e. interstitial flow or collagen stiffness) gradients^21^. Moreover, stromal cells, such as macrophages or fibroblasts, localized to specific regions within the tumor microenvironment can generate interfering signaling cues and chemoattractant gradients, which make it especially challenging, to elucidate the sources that trigger cancer cell invasion^22^,^23^,^24^.

Conventional *in vitro* 2D assays have been extensively used to assess the role of chemoattractants on cancer cell migration^25^,^26^. Wang *et al*. utilized a 2D platform simplifying the tumor microenvironment to only a monolayer of cells. They demonstrated that breast adenocarcinoma cell line (MDA-MB-231) migrated on 2D surfaces towards higher concentrations of EGF with varying speeds^25^. Although the findings were significant, the 2D model utilized in this study did not recapitulate the organization of tumor-stroma and ECM heterogeneity, normally found within the native tumor microenvironment. Most importantly, the lack of encapsulated cells within 3D ECM-based matrices, which are representative of cancer invasion within the stroma, could influence the biological findings^27^.

Traditional 3D systems currently used in invasion studies, such as Boyden chambers and Transwell migration assays, lack the ability to precisely control the spatial organization of cells in 3D matrices, cell-cell as well as cell-ECM interactions. To that end, microfluidic platforms have shown significant promise to study different aspects of cancer through better recapitulation and direct control over distinct components (i.e. cells, ECM, soluble factors) within the tumor microenvironment^28^. These recent innovations have enabled the capture of different facets of metastasis such as biochemical signaling^29^, tumor-stroma interactions^30^, invasion^31^,^32^, intravasation^33^, and extravasation^34^,^35^. Despite significant progress, most of the previous findings have relied on models with simplified or no compartmentalization of 3D interconnected tumor and stroma regions to precisely control spatial cell-cell and cell-ECM interactions. For example, some models that seeded cancer cells on 2D channels lacked the physiologically relevant influence of the 3D matrices^33^,^35^,^36^. There are also microfluidic devices that have demonstrated cell migration within 3D hydrogels^31^,^32^, but they were missing incorporation of tumor-stroma entities, thus having only a single compartment for cancer cell encapsulation. These limitations reduced their ability to study cancer invasion through side-by-side heterogeneous ECM, organized co- or tri-culture of cells as well as transport of growth factors and nutrients through diffusive barriers (i.e. solid tumors). Apart from technological development, these models often have been limited by the lack of 3D real-time single cell migration analysis from the tumor toward the stroma region during active invasion. Alternatively, models that compartmentalized cells into separated 3D stroma and tumor regions were not specifically intended to demonstrate real-time 3D invasion studies and enable modulation of microenvironmental cues (e.g. biophysical and biochemical signaling). Moreover, these platforms did not contain perfusable channels surrounding the tumor-stroma regions to assess the influence of chemotactic gradients on cancer cell invasion^30^,^37^.

In this study, we have developed a new microfluidic cancer invasion platform capable of spatially organizing 3D cell-embedded hydrogel matrices while enabling real-time capture of 3D cancer invasion within heterogeneous ECMs. The microengineered platform was designed to introduce 3D interconnected tumor and stroma regions with different ECM and cell compositions. This approach primarily provides an advantage over 2D platforms by facilitating the study of cellular migration within a native-like 3D tumor microenvironment using hydrogel-based matrices. Moreover, an important feature of our design allows the capture of an ECM-embedded high-density cell population (~15 million cells/mL) that better mimics a 3D solid tumor with dynamic diffusion of soluble factors and paracrine signaling not possible in 2D platforms^38^. On the other hand, when compared to previously established 3D platforms^30^,^31^,^33^, our model contains a specific tumor region surrounded by a separate ECM-filled entity (stromal region) permitting a juxtaposition of different ECM or cell mixtures (e.g. cancer-associated fibroblasts (CAFs)) to recapitulate cancer invasion throughout a heterogeneous tumor-stroma microenvironment. We utilized the proposed model to specifically assess how exposure to EGF impacts 3D cancer cell invasion through the stroma. In addition, the proposed design enabled generation of differential microenvironments, due to the presence of two separated channels, to specifically assess the effect of distinct gradients of EGF on cancer cell invasion. We took advantage of our device to visualize and quantify 3D cell migration metrics and morphology, utilizing advanced microscopy techniques, at both a global and single-cell level. As a result, we noted enhanced cell speed and persistence in the presence of EGF along with increased levels of cell proliferation and clustering of EGF receptors (EGFRs) indicative of cells responding to EGF. Furthermore, we correlated the enhanced invasiveness to cell morphology changes such as increased aspect ratio and number of protrusions. Overall, this work underscores a technical advance that was designed to recapitulate invasion of cancer cells in adjacent tumor and stroma regions of different ECM and cell compositions within a microengineered platform. This will allow future studies to assess the influence of various combinations of cell-cell and cell-ECM interactions on cancer invasion in a well-controlled experimental condition.

## Results

### Development of the 3D Spatially Organized Cancer Invasion Platform

**Figure 1:**
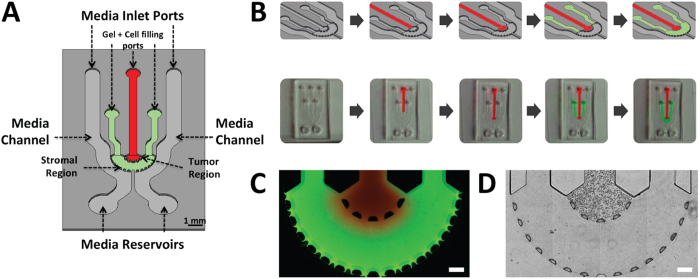
Spatial-organization of ECM and cells. (**A**) The tumor region is represented by the red color and the stroma is represented by the green. The depth of the channels is 200 μm. (**B**) Spatial-organization of cells and/or ECM is performed in a two-step process. Cancer cells were encapsulated in a tumor matrix and injected into the tumor filling port. The matrix was polymerized and then the stroma matrix was injected into the stromal filling port. This matrix was then polymerized and the final result is a tumor surrounded by a stroma. (**C**) Fluorescent image demonstrating ECM compartmentalization. A red hydrogel was injected into the tumor region while a green gel was injected into the stroma (scale bar: 200 μm). (**D**) Phase-contrast image of cells isolated within the tumor region while a cell-free stroma hydrogel surrounds it (scale bar: 200 μm).

Motivated by the need to simulate the invasion of cancer cells from the primary ‘tumor’ into the enclosing stroma, a microfluidic platform was designed to organize the cancer cells into a central tumor region surrounded by a stroma entity^39^,^40^. The microfluidic device specifically consisted of an inner chamber (tumor region) bordered by an outer chamber (stromal region) (Fig. 1A) to produce a side-by-side arrangement of 3D matrices. The diameter and height of these concentric chambers were 3 mm and 200 μm, respectively. The distance between the edge of the inner chamber and outer chamber was 1 mm. Both of these chambers were bounded by trapezoidal micro-posts spaced evenly at 100 μm to create a concentric spatial organization with clear interfaces while allowing interaction between the different regions. The trapezoid shape was chosen to permit uniform gel formation between the posts due to the angle of the trapezoid being supplementary to the contact angle of the gel solution and PDMS^41^,^42^. Tumor cells were encapsulated within a hydrogel solution and loaded first into the device through the central cell filling port (Fig. 1B and Supplementary Fig. S1). The stromal region was then loaded with cell-free hydrogel to produce an interconnected platform. The platform had two inputs that can be filled with cell media containing a variety of molecules (e.g., EGF) to enhance or suppress cancer invasion. Additionally, the design of the platform enabled creating symmetric and asymmetric gradients of biomolecules. Compared to 2D models^25^,^26^ and other conventional 3D models^31^,^33^,^38^, this assay particularly allowed for control over 3D spatial compartmentalization of cells, ECM heterogeneity, and biomolecular gradients.

To demonstrate the formation of a clear-cut spatial arrangement of ECM, we first utilized fluorescent-labeled hydrogels. Rhodamine-6G hydrogel solution was loaded into the tumor region followed by a fluorescein hydrogel solution loaded into the stromal region. After gelation, the formation of the hydrogels within specific regions of the platform was visualized under a fluorescent microscope (Fig. 1C). There were no gaps between the posts that could disrupt the interconnectivity of the two side-by-side regions, and the trapezoidal posts held the gels in place while also separating the inner and outer chambers. In that regard, the interconnectivity of the regions within the platform enabled signaling from the channels to the stroma to the tumor. Next, to demonstrate cell-ECM spatial organization, SUM-159 breast cancer cells were encapsulated within a collagen I matrix and loaded into the tumor region while cell-free collagen I was loaded into the stromal region. Following successful gelation of the hydrogels, carcinoma cells were present only within the tumor region while leaving the stroma pristine (Fig. 1D). Additionally, no gaps or bubbles were present between the tumor and stroma and the cells were distributed in 3D (Supplementary Fig. S2). The spatially organized tumor-stroma potentially allows for further studies utilizing different cell types and/or different ECM compositions, which will provide insight and understanding into how other cell types and environments influence cancer invasion^9^.

### Characterization of Diffusion Across the Platform

**Figure 2:**
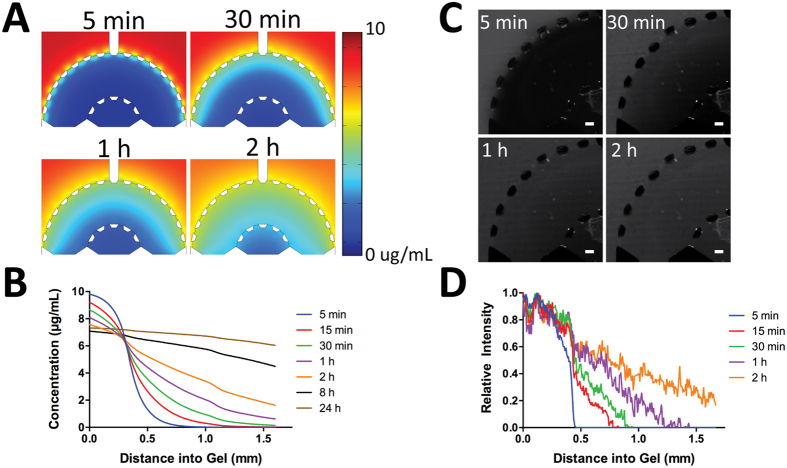
Diffusion of molecules through the two regions. (**A,B**) COMSOL simulation of 10 kDa molecule (10 μg/mL) through the device demonstrates the concentration gradient profile over time. (**C**) FITC-Dextran (10 kDa, 10 μg/mL) was diffused from the stroma to the tumor region to demonstrate successful interconnectivity and concentration gradient between the two regions (scale bar: 100 μm). (**D**) Quantified experimental results of diffusion of FITC-Dextran (10 kDa, 10 μg/mL).

COMSOL simulation of EGF diffusion was used to characterize the time-dependent gradient during chemoinvasion within the platform. Diffusion of 10 kDa molecules was simulated within a 3D collagen I gel (2.0 mg/mL) at 37 °C. Figure 2A demonstrates the computed time-lapse of the concentration gradient across the tumor-stroma microenvironment. In addition, Fig. 2B shows the simulated concentration profile within the device. To experimentally demonstrate diffusion across the platform and show that the regions within the device were well interconnected, 10 kDa FITC-Dextran (10 μg/mL) was injected into the media inlets and the resulting fluorescent intensity across the platform was recorded over time. By 30 min (Fig. 2C), the fluorescent dextran had continuously diffused across the stroma and into the tumor region, demonstrating that the interface between the two regions enabled diffusion of biomolecules from one region to the other while showing an established concentration gradient. Figure 2D provides the quantified experimental results for the concentration gradient profile showing similarities to the simulated gradient profile up to 2 h (Fig. 2B). Differences between the experiments and simulation likely arise from small convective flows generated during media changes. A gradient was established for up to 2 h and leveled out afterwards. This suggests that there is an optimum window to replenish the media at this time but other factors that can perturb the gradient profile are also in play such as cell-ECM interactions and cell-biochemical cue (i.e. the diffused cues) interactions.

### Cell Behavior within a Physiologically Relevant 3D Microenvironment

**Figure 3:**
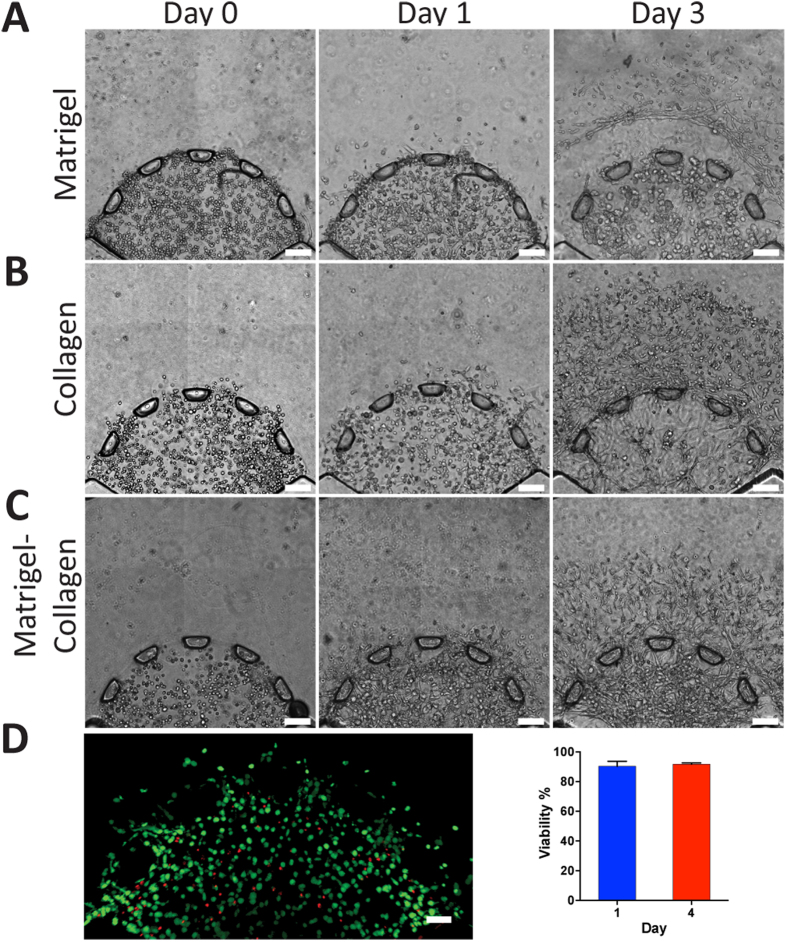
Behavior of breast cancer cells within different ECMs. (**A**) SUM-159 breast cancer cells were initially encapsulated in Matrigel^®^. On the first day, the cells demonstrated round morphology and by the third day, the matrix was disrupted (scale bar: 100 μm). (**B**) Collagen I (2.0 mg/mL) was utilized to encapsulate cells within the tumor region. The cells demonstrated similar morphology to Matrigel^®^ on the first day. On day 3, cells migrated outside of the tumor region but also exhibited great disruption of the ECM, similarly to Matrigel^®^ (scale bar: 100 μm). (**C**) A mixed gel of Matrigel^®^:collagen I (1:1 ratio) was utilized to reduce matrix disruptions. By day 3, the cells successfully migrated out but did not disrupt the tumor as heavily (scale bar: 100 μm). (**D**) LIVE/DEAD was utilized to assess the viability of the cells on day 1 and 4 within the mixed gel. The cells were highly viable and did not demonstrate any significant change between the two days (*p* < 0.05 calculated from Student’s *t*-test with more than three devices for each condition, scale bar: 100 μm).

To represent the invasive tumor microenvironment, SUM-159 breast carcinoma cells were to initially encapsulated at a high density (15 × 10^6^ cells/mL) within three distinct matrices namely, Matrigel^®^, collagen I (2 mg/mL), and 1:1 mixture of Matrigel^®^ and collagen I (final concentration of collagen I at 1 mg/mL) and were subsequently loaded into the tumor region. The stroma region was kept constant for all conditions by only loading the outer chamber with collagen I (2 mg/mL), which is the most abundant ECM protein outside the primary breast tumor area^19^,^20^. Initial studies revealed that the SUM-159 cells that were encapsulated within the Matrigel^®^ matrix demonstrated a mixture of round and elongated morphology after one day of culture (Fig. 3A). As the cells migrated out from the tumor region and into the collagen matrix of the stroma, they began to adopt more elongated morphology representative of an invasive phenotype^30^. On the other hand, breast carcinoma cells that were still within the tumor region (Matrigel^®^) maintained their roundness. SUM-159 that were encapsulated within collagen I (2 mg/mL) matrix also exhibited a mixture of round cells and elongated cells after one day of culture (Fig. 3B). However, by the third day within the collagen-only tumor region, there were little to no round cells (Fig. 3B), whereas the Matrigel^®^-only matrix still had round cells by day 3 (Fig. 3A). Although the cells encapsulated in either collagen I or Matrigel^®^ were able to successfully migrate out of the tumor region, there were significant matrix disruptions. The disruptions near the microposts created gaps within the matrix that would have prevented cells from traversing through the 3D microenvironment. To prevent the gap formation so that the cells may continuously invade from the tumor into the stroma while maintaining a physiologically relevant platform, Matrigel^®^ and collagen I (2 mg/mL) were mixed at a ratio of 1:1 prior to cell encapsulation. As seen in Fig. 3C, cells demonstrated a mixture of round and elongated morphology within the mixed gel that was similar to the previous two matrices (Fig. 3A,B) demonstrating the continuous invasion. By the third day, the cells invaded into the stroma without disruption of the matrix in contrast to the previous two conditions (Fig. 3A,B). Additionally, unlike the collagen-only hydrogel, there were still round and elongated cells within the tumor region on day 3, which demonstrated similar morphology to the Matrigel^®^-only matrix. Thus the mixed gel was chosen for all subsequent experiments to represent a more physiologically relevant primary tumor region^38^, while still allowing cells to continuously invade throughout the platform with little matrix disruption. This potential to decouple and modulate the tumor and stroma characteristics could allow further studies to develop stiffness and ligand-density gradients to elucidate the effects of haptotactic and biophysical cues on invasion dynamics.

To investigate the survival of the breast cancer cells within the mixed gel (Matrigel^®^:collagen I) microenvironment, the encapsulated cells were stained with the LIVE/DEAD assay at days 1 and 3. The live cells, which fluoresced green, and the dead cells, which fluoresced red, were counted and compared between the two days (Fig. 3D). The viability was quantified to be 90.3 ± 3.44% and 91.7 ± 0.88% for days 1 and 3 respectively and no statistical difference over the three days. These results suggested that the encapsulation of the cells within the mixed gel did not create a significant impact on cell survival.

### Tumor Growth and Dissemination of Invasive Breast Cancer Cells

**Figure 4:**
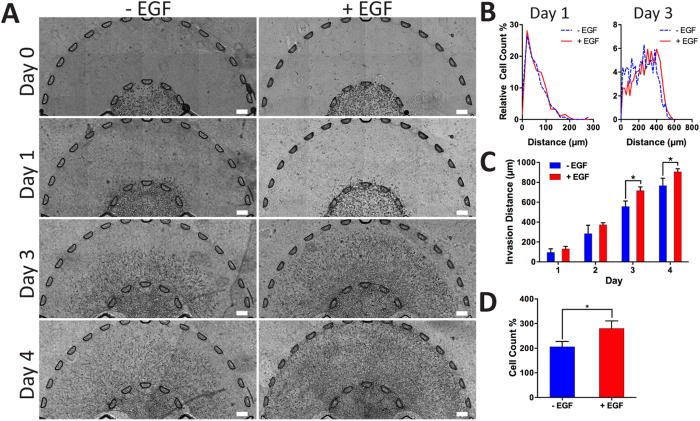
Breast cancer 3D invasion assay. (**A)** Devices were split into two groups where one group (+EGF) was introduced to EGF (50 ng/mL) and the other was not exposed to EGF (− EGF). Based on the representative images, (+) EGF showed more proliferation, as indicated by the higher density, and migration by reaching to the outer channels in 4 days. The (−) EGF group demonstrated migratory tendencies but did not have the same invasive profile as (+) EGF. (**B**) Distribution of cells was quantified for days 1 and 3. The profiles were similar between (−) and (+) EGF for the first day. By the third day, there was a shift in cell distribution toward the right indicating a higher cell count further away from the tumor region. It was not possible to quantify the distribution for the fourth day due to the high density and overlapping cells (scale bar: 100 μm). (**C**) Invasion distance of the tumor front was calculated from the radial distances of the furthest cells from the tumor region. (+) EGF exhibited significantly higher invasion by days 3 and 4 (*p* < 0.05 calculated from Student’s *t*-test with more than three devices for each condition). (**D**) Number of cells of cells was quantified on days 1 to 3 with (+) EGF leading to a significantly higher cell count (*p* < 0.05 calculated from Student’s *t*-test with more than three devices for each condition).

To understand the effects of EGF on the invasion of the SUM-159 within the spatially organized microenvironment, medium containing EGF (50 ng/mL) was added to the platform daily after an initial 24 h culturing period. The stimulated ((+) EGF) and unstimulated ((−) EGF) cells were tracked for a total of four days (96 h). During the first 24 h (Day 0), the cells adopted round morphologies and were contained within the tumor region (Fig. 4A). After one day, the cells began to invade into the stroma with a mixture of round and elongated morphologies. Cells were distributed spatially similarly between the two EGF conditions (Fig. 4B). By day 3 there was a significant difference in tumor dissemination when comparing the stimulated (715 ± 38.0 μm) and unstimulated conditions (556 ± 24.7 μm), demonstrated by a rightward distribution shift by (+) EGF cells along the x-axis (Fig. 4B). We also calculated the progress of the tumor invasion front over the four day period for both conditions and found that by days 3 and 4, the (+) EGF tumor invaded further than (−) EGF (Fig. 4C). The invading distance of furthest distal cells from the microposts (Fig. 4C) were compared daily demonstrating that by days 3 and 4, the (+) EGF tumor invaded further than (−) EGF. Moreover, whole device images taken of the actin cytoskeleton (Supplementary Fig. S3) displayed the stark difference between the two conditions in terms of tumor growth and cell invasion. The stimulated cells migrated beyond the stroma and into channels by day 4. In addition, we compared the migration of invasive SUM-159 breast cancer cells to the less invasive MCF-7 cell line within our platform. MCF-7 cells developed into clusters of cells near the edge of the tumor region (Supplementary Fig. S4A). EGF stimulation enhanced invasion of MCF-7 cells into the stroma (80 ± 8 μm) compared to the control condition without EGF (20 ± 2 μm) on day 2 (Supplementary Fig. S4B). However, when comparing to SUM-159 cells, MCF-7 invasion in presence of EGF was significantly lower by almost 10-fold within each day of the experiment (Supplementary Fig. S4C). We also investigated the effect of EGF on proliferation of SUM-159 cells by quantifying the number of cells within the stromal region which revealed increased cell count in the presence of EGF stimulation from day 0 to day 3 (Fig. 4D and Supplementary Fig. S5A). Moreover, in the (+) EGF condition expression of the proliferative marker, Ki-67, was increased (Supplementary Fig. S5B). These findings demonstrated that EGF enhances breast cancer cell invasion within the microengineered 3D platform, consistent with previous studies using 2D assays^25^.

**Figure 5:**
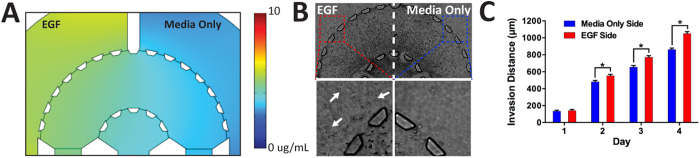
Breast cancer 3D invasion assay using asymmetric gradients. (**A**) COMSOL simulation(24 h) of 10 kDa molecule (10 μg/mL) from a single channel through the device demonstrates the concentrationgradient. (**B**) EGF (50 ng/mL) to a single channel while the other contained no supplemented EGF media. We found increased cell gravitation toward the side with EGF (**C**) Invasion distance of the tumor front was calculated from the radial distances of the furthest cells from the tumor region. (+) EGF exhibited significantly higher invasion by days 2, 3, and 4 (*p* < 0.05 calculated from Student’s *t*-test with more than three devices for each condition).

In addition to global diffusion through the platform from both media channels, we introduced EGF (50 ng/mL) to a single media channel to localize the chemoattractants asymmetrically through half of the device (Fig. 5A). The cells were consistently tracked for four days (96 h) (Fig. 5B). We observed no differences in cell migration during the first 24 h of culture. However, by day 2 there was a significant increase in invasion toward the channel in which we introduced EGF (Fig. 5C). Such trend was consistent during days 3 and 4. In fact, by day 4, there were cells that had migrated out of the stroma and into the media channels on the EGF side, however, such behavior was not observed on the other side of the stroma (the media only side channel with no EGF (Fig. 5B)). These observations clearly demonstrate the capability of our microfluidic platform to generate competing microenvironments (Fig. 5C) through introduction of variable regimes of growth factors for invasion studies.

### High Resolution Real-time Imaging of Three-dimensional Cancer Invasion at a Single-cell Level

**Figure 6:**
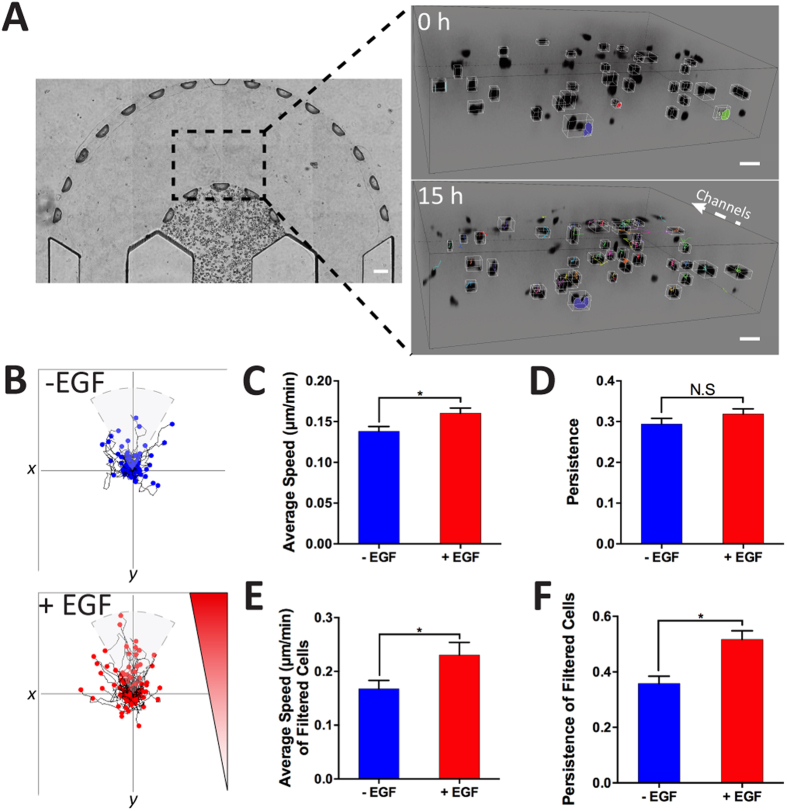
Time-lapse analysis of individually invading cells within 24 h of adding EGF. (**A**) Dashed box indicates region of interest for time-lapse capture of cell invasion. Frames from a cell invasion movie where the cells are invading throughout the stroma toward the channels (scale bar: 100 μm). **(B**) The cell trajectories were plotted. (**C**) (+) EGF cells demonstrated a significant increase in average cell speed but (**D**) no significant difference for persistence (*p* < 0.05 calculated from Student’s *t*-test with n > 50 from more than three devices for each condition). (**E**,**F**) The cells were filtered out for cells following the gradient (y-axis). We found significant increases in cell speed and persistence for (+) EGF (*p*  < 0.05 calculated from Student’s *t*-test with n > 25 from more than three devices for each condition).

**Figure 7:**
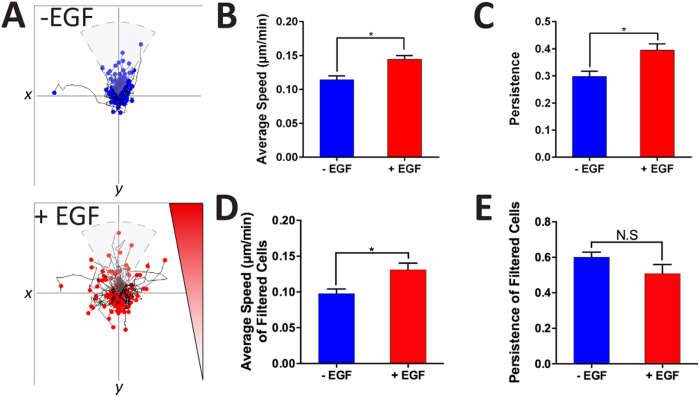
Time-lapse analysis of individually invading cells between 72 h and 96 h of EGF addition. (**A**) The cell trajectories were plotted. (**B,C**) (+) EGF cells demonstrated a significant increase in average cell speed and persistence (*p* < 0.05 calculated from Student’s *t*-test with n > 50 from more than three devices for each condition). (**D,E**) The cells were filtered out for cells following the gradient (y-axis). We found significant increases in cell speed and but no significant difference in persistence for (+) EGF (*p*  < 0.05 calculated from Student’s *t*-test with n > 9 from more than three devices for each condition).

In comparison to previous real-time migration studies^31^,^43^, our model and technique enabled us to observe the changing morphology of the invading cancer cells at a single-cell level within the 3D matrices, at a high-resolution, using light and fluorescent microscopy. In particular, cells migrated by utilizing thin protrusions probing in front of their cell body and moving their body toward the direction of the protrusions (Fig. 6A and Supplementary Movies S1 and S2). Furthermore, these cells appeared to drag their bodies along as the protrusive front moves forward. Using 3D time-lapse imaging (Supplementary Movie S3), we tracked individual cells, which was further analyzed in NIS Elements AR Microscope Imaging Software by Nikon, to investigate the effects of EGF on 3D tumor cell migration within the initial 24 h of adding EGF (Fig. 6B). We found a significant increase in average individual cell speed (Fig. 6C) (0.16 ± 0.006 *μ*m/min) compared to the (−) EGF (0.14 ± 0.006 *μ*m/min) condition. Moreover, we did not see a significant difference in the persistence (Fig. 6D) for (+) and (−) EGF at 0.32 ± 0.01 and 0.29 ± 0.01 respectively. However, when observing the cell trajectory plots (Fig. 6B), we noticed migration differences between the cells along the gradient (Fig. 2), where there were increased cell counts near the y-axis (Fig. 6B, within the pie slice). To investigate this phenomenon, we filtered the cells to only those migrating within ±30° of the gradient (y-axis) in order to isolate the sub-population of cells based on the diffusion gradient (Fig. 6A) and subsequently compare the average speed and persistence. In doing so, we found that the average speed among filtered cells was still significantly higher for (+) EGF (0.23 ± 0.02 *μ*m/min) compared to (−) EGF (0.17 ± 0.02 *μ*m/min). Furthermore, persistence within the filtered cells was found to be significantly higher for (+) EGF (0.52 ± 0.03) than (−) EGF (0.36 ± 0.03). We continued to culture the cells for two more days with daily media exchanges. The experiment was repeated again between 72 h and 96 h of culture (Fig. 7A). Consistently, we found a significant increase in average speed of individual cells (Fig. 7B) for (+) EGF (0.14 ± 0.005 *μ*m/min) compared to (−) EGF (0.11 ± 0.005 *μ*m/min) suggesting that EGF maintained high cell motility over prolonged exposure. Moreover, we noticed a significant difference in the persistence (Fig. 7C) for (+) and (−) EGF at 0.40 ± 0.02 and 0.29 ± 0.02 respectively. When looking at the filtered cells (Fig. 7D), we still found that the average speed was significantly higher for (+) EGF at 0.13 ± 0.009 *μ*m/min whereas (−) EGF demonstrated 0.10 ± 0.006 *μ*m/min. However, the difference in persistence among the filtered cells (Fig. 7E) was not found to be significant (0.60 ± 0.03 vs. 0.51 ± 0.05) suggesting cells behaving in more of a random walk fashion, at longer duration of culture, as compared to the initial 24 h of EGF stimulation.

### Analysis of EGF Receptors After Long-Term Stimulation

**Figure 8:**
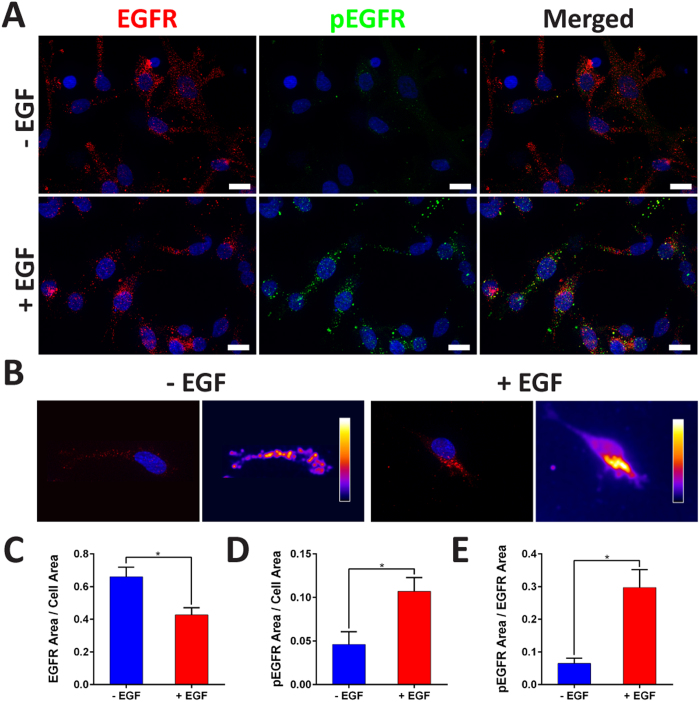
Investigation of EGFR and pEGFR. (**A**) Cells were stained for EGFR (red), pEGFR (green), and nuclei (blue) (scale bar: 20 μm). (**B**) Representative images of EGFR clusters with corresponding heat maps of relative intensities. (**C**) (+) EGF demonstrated significantly lower EGFR to cell area ratio. (**D**,**E**) (+) EGF displayed significantly higher pEGFR to cell area ratio and pEGFR to EGFR area ratio (*p* < 0.05 calculated from Student’s *t*-test with n > 18 from more than three devices for each condition).

We stained the cells for EGFRs and phosphorylated EGFRs (pEGFRs) after 1, 2, and 4 days of culture in order to visualize the patterning and activation of EGFRs in response to EGF stimulation within the device. On the first day of culture on both a 2D surface and within the hydrogel matrices (no EGF), EGFRs localized near the cell membrane (Supplementary Fig. S6A). After treatment with EGF for 24 h (Day 2 of culture, Supplementary Fig. S6B), we noticed that in the (+) EGF condition, EGFRs began to displace from the membrane toward the cell body and the distribution became more punctate^44^,^45^. In the (−) EGF condition (Day 2), the location of the EGFRs remained similar to the receptors in Day 1 images (Supplementary Fig. S6B). However, after 4 days, EGFRs no longer bordered the cell membrane, unlike the first days of culture (Supplementary Fig. S6B). Moreover, the receptors appeared in a punctate pattern throughout the cell body for both (±) EGF conditions (Fig. 8A). On the other hand, the pEGFR staining demonstrated higher intensity and a greater number of spots in the (+) EGF condition compared to the (−) EGF samples. As can be also seen in Fig. 8B, heat maps of relative surface intensity displayed high clustering of the EGFRs near the nucleus in (+) EGF condition while EGFRs were seen to be distributed through the cell body in (−) EGFs condition. We normalized the EGFR coverage area to the area of the cell body using actin as a marker for total cell area on Day 4. The EGFR to cell area ratio (Fig. 8C) was significantly higher in the (−) EGF group consistent with the clustered or localized EGFRs in (+) EGF condition suggestive of EGFR trafficking. On the other hand, the pEGFR to cell area ratio (Fig. 8D) was significantly higher in the (+) EGF group. Furthermore, for the (+) EGF group (Fig. 8E), we found a significantly higher pEGFR to EGFR area ratio suggesting more phosphorylated receptors among the available receptors. Overall, our findings suggest that the migratory phenotype of breast cancer cells, is linked to the activation of EGFRs and further demonstrates successful delivery of EGF to the cells within the 3D microfluidic device.

### Cytoskeleton Organization of Invasive Cells

**Figure 9:**
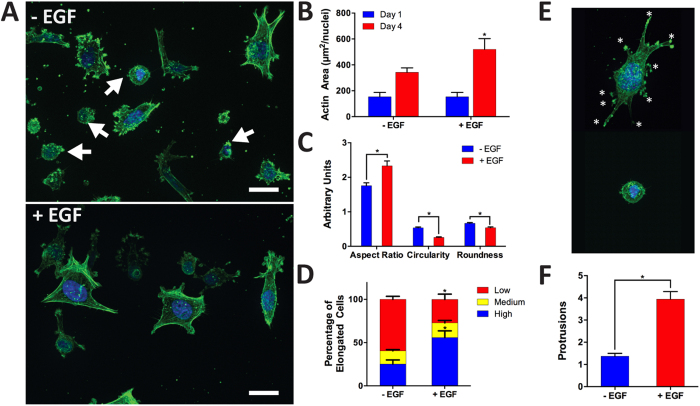
Analysis of cell morphology. (**A**) Cells were stained for F-actin (green) and nuclei (blue). (−) EGF exhibited more cells with round morphology (white arrows), which was correlated to a less invasive phenotype. (+) EGF appeared to be more protrusive (scale bar: 20 μm). (**B**) Actin area calculated from thresholded images indicated higher quantities for (+) EGF after 4 days of culture. There was no significant difference between days 1 and 4 for (−) EGF (*P* < 0.05 calculated from multiple comparisons test with more than three devices for each condition). (**C**) Cell shape descriptors of the actin-cytoskeleton were quantified using ImageJ software suite (particle analyzer plugin). (+) EGF had significantly higher aspect ratio (AR) and lower circularity and roundness; all of which correlate to a higher invasive phenotype (*p* < 0.05 calculated from Student’s *t*-test with n > 70 from more than three devices for each condition). (**D**) AR was reorganized into three categories, low: 1 < AR < 1.5, medium: 1.5 < AR < 2.0, and high 2.0 < AR. Interestingly, (+) EGF contained significantly higher amounts of cells with high ARs whereas (−) EGF contained significantly higher amounts of cells with low ARs. Both groups had no significant difference for the medium category (*p* < 0.05 calculated from Student’s *t*-test with n > 70 from more than three devices for each condition). (**E**) Protrusions (white star) were counted for each cell for the two conditions. (**F**) (+) EGF cells had significantly more protrusions than (−) EGF (*p*  < 0.05 calculated from Student’s *t*-test with n > 70 from more than three devices for each condition).

We investigated the changes in the cytoskeleton due to EGF-induced invasion. Upon close inspection we found that (−) EGF cells were not as spread as the cells within the EGF-stimulated devices. As seen in the Z-projection of the F-actin (Fig. 9A), more round cells, indicated by the white arrows, were apparent in the (−) EGF group. The actin area, which delineates the area of the cell, (Fig. 9B) was calculated by thresholding the fluorescent images and dividing the total actin area by the number of cells present in the field of view. This revealed that (+) EGF cells had almost twice the actin area than (−) EGF cells, which is representative of the higher spreading and elongation of cells seen in Fig. 9B. As previously reported, these cytoskeletal arrangements and changes in morphology are indicative of a shift towards an invasive phenotype^46^,^47^,^48^,^49^. Furthermore, specific morphology descriptors (Fig. 9C) including, aspect ratio (AR), circularity, and roundness, were calculated to divulge the effect of EGF on the cell shape. We limited the analysis to cells that were at the center of the z-stack to avoid the influence of attachment to the top and bottom of the chamber. The ARs of the cells, which indicated polarized length and extension, were significantly higher in (+) EGF (2.33 ± 0.14) than in (−) EGF (1.75 ± 0.08). The extended morphology suggested that EGF played a role in enhancing the migratory phenotype of the breast cancer cells^30^,^50^. Furthermore, the circularity of the cells within the EGF-stimulated devices was nearly half (0.25 ± 0.02) of the circularity for the (−) EGF cells (0.53 ± 0.02). These findings suggested that the protrusiveness or cell extensions were much lower in unstimulated cells. Moreover, the roundness of the unstimulated cells was 0.66 ± 0.02 compared to 0.53 ± 0.02, which reflected the significantly higher amount of round cells for (−) EGF demonstrated in Fig. 9A. Interestingly, the distribution of elongated cells were opposite for the two conditions (Fig. 9D). Specifically, the ARs of the cells were binned into three different groups, high (AR > 2), medium (2 > AR > 1.5), and low (1.5 > AR). As can be seen in Fig. 9D, 55.8 ± 7.72% of (+) EGF cells were regarded as high, whereas only 25.2 ± 4.73% of (−) EGF had high ARs. In contrast, (−) EGF showed the enhanced low ARs and reduced high ARs. For the (−) EGF condition, 59.6 ± 3.49% of the cells had low ARs in comparison to the 27.2 ± 6.13% of stimulated cells. There was no significant difference between the medium AR results. Furthermore, we examined the effect of (−) EGF and (+) EGF on protrusiveness (Fig. 9E). On average, (+) EGF exhibited 3.94 ± 0.34 protrusions per cell whereas (−) EGF demonstrated 1.36 ± 0.13 (Fig. 9F). Furthermore, the protrusion count per cell was compared with the aspect ratio and the circularity to determine the relationship between cell protrusion and cell morphology under the context of EGF stimulation. These results suggested that ARs and cell protrusions slightly correlated (R-squared = 0.11, *p* < 0.001) prior to EGF stimulation, but upon introduction of EGF the correlation was negligible (R-squared = 0.00, *p* < 0.61). Furthermore, protrusions were correlated with circularity (Supplementary Fig. S7) indicating that under (−) EGF condition, the circularity sharply decreased when cell protrusions increased. With EGF stimulation, circularity moderately declined (R-squared = 0.16, *p* < 0.001) with increasing cell extensions where the correlation was weaker than (−) EGF condition (R-squared = 0.45, *p* < 0.0001). This result suggested that EGF stimulation reduced the correlation between circularity and cell protrusions similarly when looking at ARs and protrusions.

**Figure 10:**
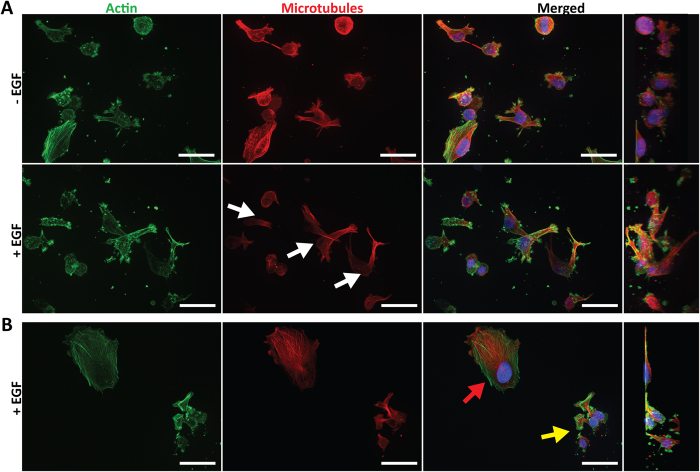
3D Analysis of F-actin and microtubules. (**A**) Fluorescent staining revealed cell morphology within the 3D matrix. Cells appeared to be round with slight protrusions. Microtubule fluorescence was intense in all directions for (−) EGF, but had a slight affinity toward a single direction (white arrows) in the (+) EGF group. (**B**) Cell on 2D substrate appeared to be flat comprised of wide lamellipodia (red arrow). Cells moving toward the glass substrate had round cell body but large and wide protrusions (yellow arrow).

Further immunostaining, clearly demonstrated microtubule networks in both conditions (Fig. 10A). We also observed morphology changes between 3D stroma and 2D glass surface. In Fig. 10B, the red arrow indicates a cell migrating across the 2D plane while the yellow arrow indicates cells exiting the stroma. The morphology of the cells on 2D plane (Fig. 10B, red arrow) was distinctly different from that of the cells encapsulated within the 3D stroma (Fig. 10A and Supplementary Movies S4 and S5) showing that the device can recapitulate 2D and 3D morphologies^51^,^52^. The actin stress fibers (Fig. 10B, red arrow) spread across the relatively flat cell body with partial local alignment toward the direction of movement. Furthermore, the microtubules exhibited higher amount of fibers and polarization in the same direction. When observing the cells in the 3D stroma (Fig. 10A and Supplementary Movie S4 and S5), the cells were less spread, more spherical, and either with or without protrusions. The cells within the stroma presented long protrusions reaching into the matrix whereas cells on the 2D surface had short and flat protrusions (Supplementary Fig. S8, white and yellow arrow indicates protrusion and collagen stroma respectively, Supplementary Movie S6). Additionally, in Fig. 10B, the cells transitioning out of the matrix demonstrated a spherical cell body, similar to cells embedded within the 3D stroma. The flat short protrusions that were reaching out onto the 2D surface were characteristics of cells on glass substrate.

### CAFs Enhance Breast Cancer Cell Invasion

**Figure 11:**
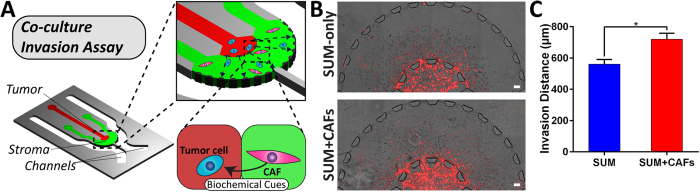
(**A**) Schematic of tumor cell and CAF interactions within the 3D platform. (**B**) SUM-159 cells expressing mCherry (red) invading into the stroma with or without CAFs, scale bar: 100 μm. (**C**) Comparison of migration between SUM-159 only and SUM-159 + CAFs groups on day 3. (*p*  < 0.05 calculated from Student’s *t*-test with n > 6 devices for each condition).

To incorporate further complexities in the proposed tumor microenvironment, we investigated the invasive response of SUM-159 cells in the presence of CAFs (Fig. 11A). Specifically, we generated a tumor region comprised of SUM159 cells embedded in a 1:1 mixture of Matrigel^®^ and collagen I (final concentration of collagen I at 1 mg/mL) and a surrounding stromal region consisting of 100,000 cells/mL CAFs embedded in collagen I (2 mg/mL). In doing so, a 3D tumor-stroma arrangement of breast cancer cells and fibroblasts was successfully generated within the microfluidic device. Over the course of 3 days, SUM-159 cells expressing mCherry migrated into the surrounding stroma (Fig. 11B), where the presence of CAFs influenced the invasive profile of cancer cells (Fig. 11C). Specifically, CAFs induced higher invasion distance in SUM-159 cells (720 ± 15 μm) as compared to the control condition (564 ± 12 μm) containing no fibroblasts.

## Discussion

Despite significant progress, the majority of microfluidic models lack cell and ECM spatial organization that allows for separate manipulation of side-by-side tumor and stromal regions to assess the roles of microenvironmental factors on 3D cancer invasion, in a real-time fashion^25^,^26^,^28^,^53^. For instance, work done by Sung *et al*.^30^,^37^ provided 3D microenvironment models to spatially compartmentalize different cell types, however the overall objective of the work was not to assess real-time cancer cell invasion in 3D matrix. Additionally these models did not incorporate perfusable channels surrounding the tumor-stroma regions to allow for diffusion of biomolecule gradients of chemotactic factors to the cells. Instead, their model was used to show differences in cell phenotype, which we also demonstrated in our platform. Furthermore, Kim *et al*.^31^, studied invasion in a 3D hydrogel but the model did not offer the ability to separately compartmentalize cancer cells and stromal ECM. Moreover, work by Zervantonakis *et al*.^29^,^33^,^42^, provided insight on the role of tumor-macrophage-endothelial interactions in invasion within a microfluidic tumor model, but was not designed to examine side-by-side arrangement of different 3D ECM components, which is crucial in understanding the invasion process. Our model enabled compartmentalization of tumor and stroma entities, which would allow for fine-tuning and control of heterogeneous ECM and cell components, as well as diffusive barriers (i.e. solid tumors) for nutrient and growth factor transport. Another contribution of our work was real-time high-resolution 3D invasion (in all x, y, z dimensions) and morphology studies at the single cell level.

Cancer cells were compartmentalized within our platform to represent a tumor surrounded by a stromal matrix (Fig. 1) where biochemical cues (i.e. EGF) diffused from outside of the stoma toward the center of the tumor region (Fig. 2). We primarily investigated the cell morphology and tensional homeostatic effect of encapsulating SUM-159 cells within three different matrices namely Matrigel^®^, collagen I, and 1:1 mixture of Matrigel^®^ and collagen I (Fig. 3). Our analysis revealed that as the cells migrated and proliferated, they disrupted in the matrix either by proteolytic or tensional effect^54^ showing that either collagen I or Matrigel^®^ matrix alone was unable to maintain high fidelity of the tumor-stroma architecture. Furthermore, by mixing collagen I and Matrigel^®^ (1:1 ratio), the combined matrix was able to support continuous migration (from tumor to stroma) and proliferation of the cancer cells with similar morphology compared to Matrigel^®^-only matrix. Collagen I was chosen for the surrounding stroma matrix where we demonstrated that the two matrices (i.e. Matrigel^®^:collagen I at 1:1 and collagen I) were not mixed but spatially organized as a stromal (collagen I) component surrounding the tumor region (Matrigel^®^:collagen I, Fig. 1). Such capability will enable addition of other cell types (i.e. CAFs) within the stroma region, which have been known to reduce chemoresistance^37^ as well as enhance tumor growth and invasion^6^.

We modulated the invasiveness of breast cancer cells by the introduction of a 3D EGF gradient within the tumor-stroma platform. The encapsulated breast cancer cells migrated (Supplementary Movie S1) in a radial direction outward from the 3D tumor region. EGF was found to heavily influence cell proliferation and invasion distance to which by day 4, there were stark differences in tumor area coverage between the two studied conditions. The (+) EGF devices exhibited further invasion and increased transitioning from the 3D stroma to the surrounding media channels (Fig. 4). Moreover, we showed that addition of EGF increased invasion in the non-invasive cell line, MCF-7 (Supplementary Fig. S4). However when compared to SUM-159 cells, the level of invasiveness of MCF-7 cells into the collagen stroma was almost 10-fold lower, demonstrating the ability of our platform to model the 3D migratory profile of a variety of cancer cell lines. Our platform also enabled creating differential microenvironments, specifically through introducing EGF in only one of the two segregated channels. SUM-159 cells were attracted to and demonstrated higher invasion to the EGF side (Fig. 5). Our findings were consistent with those from *in vivo* studies^18^,^24^ that demonstrated EGF enhanced invasion within mouse models. However, real-time high-resolution tracking of individual cells and visualization of 3D cell morphology were not possible using *in vivo* models^18^,^38^. Moreover, in previous microfluidic models^31^,^33^ that utilized EGF as a chemoattractant, cell invasion characteristics were not fully captured within a 3D matrix in all x, y and z dimensions.

Our analysis of the real-time imaging (Supplementary Movies S2 and S3) revealed that the cells increased their individual motility in response to EGF, which confirms that the invasion of the stroma region was not limited to cell proliferation (Fig. 4D) but also included chemokinesis (Figs 6 and 7). We found that during the initial 24 h, the whole cell population responded to EGF with increased motility but the overall persistence was not significant. However, when looking only at the filtered cells migrating along the gradient (y-axis) (Fig. 6D,E), we found significant increases for individual cell motility and persistence in (+) EGF condition. As expected, there was no difference in persistence for (−) EGF for the whole population of cells as well as the filtered cells (Supplementary Fig. S9). This suggests that the population of cells may be heterogeneous in that sub-populations respond to EGF differently^55^. Therefore, by analyzing chemotactic responses based on population averages, the end results may fail to account for the aggressive sub-population that can contribute the most to invasion^56^. For longer times (after 72 h), there was no significant difference in persistence toward the gradient (Fig. 7E) which overall appeared to be more of a random walk. This suggests that over saturation of EGF (72 h) may prolong the overall persistence regardless of the direction (Fig. 7C). Moreover, it has been noted in studies utilizing 2D platforms that EGF treatment induces internalization of EGFRs through endocytosis^44^,^57^ to regulate processes such as cell migration^45^. The data presented here (Fig. 8 and Supplementary Fig. S6) demonstrate that our 3D model reiterates the current understanding of EGFR trafficking in 2D after activation with EGF. In addition, several studies have indicated that prolonged exposure to EGF, such as in our investigation, will internalize or localize clusters of EGFRs thus reducing the amount of surface EGFRs^44^,^58^ (Fig. 8). However, none of the previous studies showed prolonged loss of 3D chemotactic responses (i.e. persistence toward the gradient), despite ongoing chemokinesis (i.e. cell speed) in the later stages of invasion, which may be due to saturation of EGFRs^25^,^26^,^31^,^44^,^45^,^57^. This could be a potential area of study to investigate the prolonged spatiotemporal signaling of EGF, in relation to chemokinetic and chemotactic responses, in cancer cells.

Cell morphology analysis, indicated that cells migrating on the glass appeared to have flat and wide protrusions resembling lamellipodia. These cells (Supplementary Movie S7) appeared to follow the characteristic migration steps, which are the exploration and then attachment of the leading edge followed by the detachment and pulling of the rear cell body^59^. On the other hand, within 3D matrix, the cells did not clearly exhibit the classical stages of migration^51^ but instead appeared to entangle inside the matrix with the thin-like protrusions (Supplementary Movie S2), which induced a slow moving crawl. Similarly, F-actin staining of the encapsulated cells (Supplementary Fig. S3) revealed thin protrusions surrounding the cell body correlating to what was observed in the migrating cells (Supplementary Movie S2). Consistently, Lämmermann *et al*. demonstrated elongation and dragging of the cell body, hypothesized that this phenomenon happened in areas of increased spacing between collagen fibers^60^. Similarly to the results found with our device, Fraley *et al*. showed several protrusions extending from migrating cells in 3D matrices but did not show wide lamellipodia in 3D. Furthermore, they suggested that the cells utilized these protrusions to probe the surrounding matrix which led to correlating the extent and amount of protrusions to cell motility in 3D^61^. We further observed noticeable deformation of the collagen matrix overtime in the device, which was the driving factor to producing a suitable matrix composition for the encapsulation of the invasive cancer cells. When closely observing the migrating cells, there were some cells that tended to follow in the tracks of another cell. This observation was in line with previous work by Gaggioli *et al*. who studied invasion inducing-microtracks^62^. This behavior would require further analysis to elucidate the mechanisms of this migratory phenotype.

Stromal cells, such as fibroblasts, endothelial cells, and immune cells, have been demonstrated to heavily influence cancer invasion and therapy^38^,^63^. In particular, CAFs have been shown to enhance cancer survival and invasion through cell-cell signaling^63^. In our model, we showed that in the presence of CAFs, SUM-159 cells migrated further into the surrounding stromal region compared to the control condition (no CAFs) (Fig. 11C). Based on previous literature and our own experiments, we believe that CAFs are potentially secreting chemoattractants^64^ such as stromal cell-derived factor 1α (SDF-1α or CXCL12) that promote breast cancer invasion. This phenomenon creates a unique opportunity for future studies to mechanistically assess the influence of paracrine signaling and the resulting effects within a heterogeneous population of cells on cancer invasion using our proposed platform.

Taken together, our microfluidic platform demonstrated the capability of studying tumor growth and cancer cell migration at a single cell level with the advantage of direct control over spatial cell-ECM, cell-cell and cell-ligand interactions. In our future studies, we aim to incorporate stromal cells (i.e. cancer associated fibroblasts) surrounding the tumor region for more physiologically relevant invasion studies.

## Conclusions

In this work, a new 3D microfluidic platform, designed with separate tumor-stroma entities, was developed to recapitulate 3D cancer cell invasion. Our platform enabled, precise control over cell-ECM and cell-growth factor interactions. We specifically investigated the invasion enhancing effects of EGF and validated the platform as a real-time functional assay for fundamental biological processes (i.e. cell invasion, cellular signaling). Our unique approach allowed for visualization and quantification of invasion and morphology changes, at a single cell level, which was not possible in conventional Transwell assays, 3D macroscale hydrogels, and animal models. We spatially organized a high density of SUM-159 breast cancer cells within a confined 3D tumor region composed of Matrigel^®^ and collagen type I. A stromal matrix of collagen type I surrounded the tumor region, which allowed diffusion of EGF through the stroma into the primary tumor. On a global level, we observed enhanced invasiveness of the breast cancer tumor front when stimulated with EGF. At a single-cell level, we performed real-time 3D migration, which confirmed the increased motility and persistence of cells due to EGF stimulation. Moreover, we showed changes in persistence and migration at different time frames, where we observed that initially a subset of cells migrated preferentially toward the gradient. However, after prolonged exposure to EGF, we found no difference in persistence between the EGF condition and the control despite still increasing cell motility due to EGF. Subsequently, we found higher EGFR clustering within the cell after 4 days of EGF exposure suggesting possible receptor saturation^44^,^58^, which could explain the lowered chemotactic responses at later time points. We observed cytoskeletal and morphological changes in the EGF-stimulated devices, where the cells demonstrated a more invasive phenotype (i.e. increased aspect ratio and reduced circularity) with higher protrusion counts. Lastly, we investigated the co-culture of SUM-159 cells and CAFs within the tumor and stroma region respectively, which resulted in enhanced SUM-159 invasion. Together, this demonstrates the ability of our device to visually observe a combination of cell migration, morphology, survival, and proliferation changes, which provides a valuable tool that recapitulates 3D tumor-stroma interaction and invasion in a single platform.

Our future studies will be focused on adding complexities to this process by studying heterotypic cell interactions, such as the investigating the diverse roles of CAFs within a 3D stromal region. We further plan to take advantage of our device to investigate and image real-time changes in cell morphology and migration under different therapeutics regimens such as suberoylanilide hydroxamic acid^49^, nocodazole^48^, paclitaxel^65^, etc. Moreover, we aim to manipulate the cell-cell interactions to study how the heterotypic dialogue affects drug resistance.

## Materials and Methods

### Microfluidic design and fabrication

The microfluidic platform was fabricated using photo- and soft-lithography techniques. First, the design was created utilizing cad software, and subsequently, a transparent mask was fabricated from this design to undergo SU-8 photolithography. SU8-2075 (MicroChem) was spun onto a silicon wafer with a height of 200 μm. Next, the wafer was placed underneath the transparent mask and exposed to UV to generate the mold for the microfluidic device. Polydimethylsiloxane (PDMS, Sylgard 184 Silicon Elastomer Kit, Dow Corning) was casted over the SU-8 wafer and baked for 1 h at 80 °C. Afterwards, the PDMS was peeled off and the devices were cut out with blades, and afterwards, the inlets and outlets were cored using biopsy punches. Next, these devices were treated with oxygen plasma (PDC-32G, Harrick Plasma) and were then bonded to glass with channel side facing down to create a sealed environment. The fabricated microfluidic chips were sterilized by wet autoclave followed by a dry autoclave. The chips were surface treated by injecting 1 mg/mL poly-d-lysine (PDL, Sigma-Aldrich) into the channels and chambers. The devices were incubated at 37 °C for 1 h and then washed with deionized (DI) water. Next, 0.1% (v/v) glutaraldehyde (GA, Sigma-Aldrich) solution was added into the channels and chambers and incubated at room temperature for 1.5 h. Finally, the devices were washed 4 times with DI water and placed in an 80 °C oven overnight to render the surfaces hydrophobic.

### Diffusion across the tumor-stroma

COMSOL simulation of EGF diffusion was used to characterize and predict the time-dependent gradient within the platform. Diffusion occurs primarily in the x-y plane due to the fact that the imposed concentrations in the channels are independent of z, there is no flux through the top and bottom surfaces, and the geometry is uniform in z. Thus, EGF was uniformly distributed in the z-direction aside from small variations due to the distribution of cells within the gel regions. Note also that the diffusion coefficient of collagen is relatively constant in all directions within the hydrogel so there should be no major change in diffusion influenced in the z direction of the hydrogel. Therefore, we utilized a two-dimensional model to simulate diffusion of EGF in one x-y plane of the 3D device. Based on Stokes-Einstein 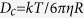 relationship^66^, where 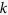 is the Boltzmann constant (1.38 × 10^−23 ^J • K^−1^), 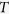 = 298.15 K, 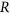 is the Stokes radius (23 angstroms), and 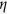 is the dynamic viscosity of the media (0.78 × 10^−3 ^N • s/m^2^)^67^, the diffusion coefficient for a 10 kDa molecule within media at 37 °C was calculated to be 9.25 × 10^−11^ m^2^/s. The coefficient for the 3D collagen I gel (2.0 mg/mL) was determined to be 8.7 × 10^−11^ m^2^/s by multiplying the media coefficient by the diffusion hindrance coefficient 0.94 (i.e. diffusion through the gel compared to the solution). This value is predicted based on the area fraction of collagen related to the fiber diameter of isotropic fiber networks. The pores (>0.75 μm for collagen area fraction of less than 1%)^68^ within the collagen hydrogel being larger than the hydrodynamic radius of the dextrans^33^,^68^ resulted in approximate diffusion hindrance to be 0.94 based on work done by Stylianopoulos *et al*. Inlet was set to zero flow, the outlet was set to zero pressure, the concentration of the molecule within the channels was set equal to 10 μg/mL, and the inlet concentrations were set constant at 10 μg/mL.

Fluorescent dextran (10 kDa FITC-Dextran (10 μg/mL)) was injected into the media inlets and the resulting fluorescent intensity across the platform was recorded over time for 2 h every 5 min. Relative intensity of the concentration gradient was quantified in ImageJ by using the profile tool.

### Cell culture

SUM-159 breast cancer cells was chosen as a suitable cell type to invade through a 3D hydrogel^69^. The MCF-7, SUM-159 and mCherry-labeled SUM-159 breast cancer cells, provided by the Mouneimne lab, were cultured in MCF-7 specific media (Dulbecco’s Modified Eagle Medium supplemented with 10% heat inactivated fetal bovine serum (FBS), 1x penicillin-streptomyocin) and SUM specific media (Ham’s F-12 with L-glutamine and supplemented with 5% heat inactivated fetal bovine serum (FBS), 1x penicillin-streptomyocin, 1 μg/ml hydrocortisone, and 5 μg/ml insulin) respectively. CAFs (passage > 55) were acquired from ATCC (ATCC^®^ HTB 125™) and cultured in MCF-7 specific media. The cells were maintained within a humidified cell culture incubator at 37 °C and 5% CO_2_.

### Invasion assay

To load the cancer cells into the device for invasion experiments, cells were trypsinized and pelleted into 0.5 mL microcentrifuge tubes. Matrigel^®^ (Corning), collagen I (2.0mg/mL, Corning^®^ Collagen I, Rat Tail, 100mg, Product #354236, Corning) and a 1:1 mixture of Matrigel^®^ to collagen type I (2.0 mg/mL) was added to the cells to create a mixed hydrogel cell solution (final concentration of collagen I at 1 mg/mL) with a density of 15 million cells/mL. We optimized the cell density ranging from 1 million/mL to 40 million/mL in order to create a dense solid tumor (estimated to be 50 to 400 million/mL from xenografts)^70^,^71^. The preliminary optimization indicated that lower densities (5 million/mL) did not allow us to obtain a good sufficient population of migrating cells within the first 4 days of experiment for subsequent analysis. The mixed hydrogel cell solution was injected into the tumor region of the microfluidic chip and polymerized by placing the devices into the humidified cell culture incubator at 37 °C. These chips were flipped every 1 min to create a homogenous distribution of cells within the mixed gel. After 8 min within the incubator, the devices were taken out and subsequently a 2.0 mg/mL collagen type I solution was injected into the stromal region. For co-culture with CAFs, 100,000 cells/mL were encapsulated in the collagen type I solution. The collagen was polymerized within the humidified incubator at 37 °C for 8 min. Next, SUM media was added into the channels of each device and the microfluidic chips were placed into the cell culture incubator overnight. On the next day, the media within EGF-stimulated devices ((+) EGF) was exchanged with SUM media supplemented with EGF (50 ng/mL). Unstimulated devices ((−) EGF) were based on the use of normal SUM media. Media was changed daily for the whole culture period.

Phase-contrast images of the cells were acquired once every day using 4 × 3 tiles with a 10x objective. Distribution of invading cells was quantified from these images by measuring the distance from the cells to the nearest micropost. If there was a cell in a cluster, only the furthest point on the furthest cell from that cluster was measured unless clear cell borders were distinguishable. Each measurement was taken from the most distal protrusion of the cell (not the cell body). To quantify the invading edge of the tumor (i.e. the invading tumor front), only the cells on the periphery were measured by considering the most distal points of the most distal cells. In this case, only the cells at the edge of the tumor (i.e. cells that have traveled the highest distance from the tumor region from each degree) were included in the quantification.

To perform time-lapse imaging, mCherry-labeled SUM-159 cells were mixed together with normal SUM-159 at a ratio of 1:9 prior to the invasion assay. The devices were placed inside a custom miniature incubator (TC-MWP, Bioscience Tools) with a 4-well insert, which enabled imaging of 4 devices at a time at 37 °C and 5% CO_2_. Fluorescent time-lapse imaging was performed using a fluorescent microscope (Zeiss Axio Observer Z1, Zeiss) equipped with the Apotome.2 and a 10x objective. The z-resolution and the time interval were set to 3.45 μmand 45 min, respectively. 3D time-lapse images were taken within 24 h of adding EGF and between days 3 and 4 of the invasion assay. Speed (defined as distance over time) and persistence (defined as euclidean distance over accumulated distance) were utilized to quantify cell migration metrics. To filter out the cells to obtain those that migrate toward the gradient, the average angles of each cell trajectory were compiled and reduced to those cells within ±30° of the y-axis (i.e. the direction of the gradient).

### Fluorescent staining

For immunofluorescent (IF) staining, the cells were fixed by adding warmed 4% paraformaldehyde into the channels of the microfluidic chip for 30 min. The devices were then washed twice with PBS-glycine (100 mM glycine in PBS) for 10 min at room temperature. This was followed by a single wash using PBS-Tween (0.05% (v/v) Tween-20 in PBS). Next, IF buffer (0.2% (v/v) Triton X-100 + 0.1% (v/v) BSA (radioimmunoassay grade) + 0.05% Tween 20, 7.7 mM NaN_3_ in PBS) + 10% (v/v) goat serum was added into the channels and the devices were incubated at room temperature for 1.5 h. Later, primary antibodies, monoclonal Anti-α-Tubulin (1:500, T9026, Sigma-Aldrich), Ki-67 (1:100, (DSHB Hybridoma Product AFFN-KI67-3E6)), EGFR (1:1000, MA5-13319, Thermo Scientific), or pEGFR (1:100, 3777S, Cell Signaling Technology^®^) were diluted at in IF buffer and devices were parafilmed and kept at 4 °C overnight. Afterwards, the microfluidic channels were washed 3 times utilizing IF buffer for 20 min each. Then, the secondary antibody (Alexa Fluor^®^ 488, 555, or 647, Thermo Fisher Scientific) was diluted in IF buffer for 45 min at room temperature in the dark. The devices were washed once with IF buffer for 20 min and twice with PBS-Tween for 10 min each at room temperature. Afterwards, the nuclei and F-actin fibers were stained by using 4′,6-diamidino-2-phenylindole (DAPI, Invitrogen) (1:1000) and Alexa Fluor^®^ 488 Phalloidin (Invitrogen) (1:40) overnight at 4 °C. The dilutions were done using PBS-Tween. Finally, the devices were washed 5 times in PBS-tween for 10 min each before imaging.

### Imaging and analysis

All imaging was performed using a Zeiss Axio Observer Z1 with the Zen Pro software suite. Phase-contrast images of the invasion assay were captured using a 10x objective lens. Immunofluorescent images were taken with 10x and 40x objective lenses together with the Apotome.2 (Zeiss). The Apotome.2 created optical sections of our devices, reducing scattered light, to generate clear fluorescent z-stacked images for 3D images.

To quantify cell proliferation, cells expressing mCherry were counted on days 1 and 3 and compared. Moreover, Ki-67 proliferative marker was also quantified by comparing the cells expressing Ki-67 to total cell population. The index was developed by dividing Ki-67 positive cells by all nuclei within a field of view (40x magnification, 223 × 167 μm^2^).

To analyze EGFRs and pEGFRs on days 2 and 4, we thresholded images (40x magnification) of the receptors in ImageJ with actin for their respective coverage areas. EGFR and pEGFR areas were divided by the area of the cell body resulting in a normalized ratio. We also divided the pEGFR area by the EGFR area to analyze the phosphorylated receptors among the non-phosphorylated ones. Heat maps were created using the 3D surface plot plugin in ImageJ.

To investigate the cytoskeletal organization of the cells, we looked at the actin area, cell shape, and protrusiveness. The actin fibers were thresholded in ImageJ producing an area value based on the number of pixels representing the F-actin fiber coverage area. Subsequently, this area was divided by the cell total count (nuclei) in the field of view (20x magnification, 0.45 × 0.34 mm^2^) on days 1 and 4 of culture. We used the particle analyzer plugin within the ImageJ suite to further quantify cell shape (i.e. aspect ratio, circularity, and roundness) based on the actin masks. Finally, the protrusions of the cells were manually counted in the actin cytoskeleton by quantifying the number of extensions (>3 μm in length and 1 μm in width) from the cell body.

### Statistical analysis

All measurements were compiled from three or more independent devices for each experimental condition. The data were compared using unpaired Student’s *t*-tests, multiple comparisons tests, and correlation analysis within the GraphPad Prism software (GraphPad Prism 6 Software).

## Additional Information

**How to cite this article**: Truong, D. *et al*. Breast Cancer Cell Invasion into a Three Dimensional Tumor-Stroma Microenvironment. *Sci. Rep.* **6**, 34094; doi: 10.1038/srep34094 (2016).

## Supplementary Material

Supplementary Information

Supplementary Movie S1

Supplementary Movie S2

Supplementary Movie S3

Supplementary Movie S4

Supplementary Movie S5

Supplementary Movie S6

Supplementary Movie S7

## References

[b1] Siegel R., Ma J., Zou Z. & Jemal A. Cancer statistics, 2014. CA Cancer J Clin 64, 9–29, 10.3322/caac.21208 (2014). 24399786

[b2] Hanahan D. & Weinberg R. A. Hallmarks of cancer: the next generation. Cell 144, 646–674, 10.1016/j.cell.2011.02.013 (2011). 21376230

[b3] Gupta G. P. & Massague J. Cancer metastasis: building a framework. Cell 127, 679–695, 10.1016/j.cell.2006.11.001 (2006). 17110329

[b4] Mao Y., Keller E. T., Garfield D. H., Shen K. & Wang J. Stromal cells in tumor microenvironment and breast cancer. Cancer Metastasis Rev 32, 303–315, 10.1007/s10555-012-9415-3 (2013). 23114846 PMC4432936

[b5] Conklin M. W. & Keely P. J. Why the stroma matters in breast cancer: insights into breast cancer patient outcomes through the examination of stromal biomarkers. Cell Adh Migr 6, 249–260, 10.4161/cam.20567 (2012). 22568982 PMC3427239

[b6] Khamis Z. I., Sahab Z. J. & Sang Q. X. Active roles of tumor stroma in breast cancer metastasis. Int J Breast Cancer 2012, 574025, 10.1155/2012/574025 (2012). 22482059 PMC3296264

[b7] Thoma C. R., Zimmermann M., Agarkova I., Kelm J. M. & Krek W. 3D cell culture systems modeling tumor growth determinants in cancer target discovery. Adv Drug Deliv Rev 69–70, 29–41, 10.1016/j.addr.2014.03.001 (2014). 24636868

[b8] Lee J. M. . A three-dimensional microenvironment alters protein expression and chemosensitivity of epithelial ovarian cancer cells *in vitro*. Laboratory Investigation; a Journal of Technical Methods and Pathology 93, 528–542, 10.1038/labinvest.2013.41 (2013). 23459371

[b9] McMillin D. W., Negri J. M. & Mitsiades C. S. The role of tumour-stromal interactions in modifying drug response: challenges and opportunities. Nat Rev Drug Discov 12, 217–228, 10.1038/nrd3870 (2013). 23449307

[b10] Paraiso K. H. T. & Smalley K. S. M. Fibroblast-mediated drug resistance in cancer. Biochemical Pharmacology 85, 1033–1041, 10.1016/j.bcp.2013.01.018 (2013). 23376122

[b11] dit Faute M. A. . Distinctive alterations of invasiveness, drug resistance and cell-cell organization in 3D-cultures of MCF-7, a human breast cancer cell line, and its multidrug resistant variant. Clin Exp Metastasis 19, 161–168 (2002). 11964080 10.1023/a:1014594825502

[b12] Knowlton S., Onal S., Yu C. H., Zhao J. J. & Tasoglu S. Bioprinting for cancer research. Trends in biotechnology 33, 504–513, 10.1016/j.tibtech.2015.06.007 (2015). 26216543

[b13] Hagemann T. . Enhanced invasiveness of breast cancer cell lines upon co-cultivation with macrophages is due to TNF-alpha dependent up-regulation of matrix metalloproteases. Carcinogenesis 25, 1543–1549, 10.1093/carcin/bgh146 (2004). 15044327

[b14] Goswami S. . Macrophages promote the invasion of breast carcinoma cells via a colony-stimulating factor-1/epidermal growth factor paracrine loop. Cancer Res 65, 5278–5283, 10.1158/0008-5472.CAN-04-1853 (2005). 15958574

[b15] Dudu V., Able R. A. Jr., Rotari V., Kong Q. & Vazquez M. Role of Epidermal Growth Factor-Triggered PI3K/Akt Signaling in the Migration of Medulloblastoma-Derived Cells. Cell Mol Bioeng 5, 502–413 (2012). 24273611 10.1007/s12195-012-0253-8 PMC3832994

[b16] Meng Q., Xia C., Fang J., Rojanasakul Y. & Jiang B. H. Role of PI3K and AKT specific isoforms in ovarian cancer cell migration, invasion and proliferation through the p70S6K1 pathway. Cell Signal 18, 2262–2271, 10.1016/j.cellsig.2006.05.019 (2006). 16839745

[b17] Price J. T., Tiganis T., Agarwal A., Djakiew D. & Thompson E. W. Epidermal growth factor promotes MDA-MB-231 breast cancer cell migration through a phosphatidylinositol 3 ’-kinase and phospholipase C-dependent mechanism. Cancer Research 59, 5475–5478 (1999). 10554021

[b18] Wyckoff J. . A paracrine loop between tumor cells and macrophages is required for tumor cell migration in mammary tumors. Cancer Res 64, 7022–7029, 10.1158/0008-5472.CAN-04-1449 (2004). 15466195

[b19] Provenzano P. P. . Collagen density promotes mammary tumor initiation and progression. BMC Med 6, 11, 10.1186/1741-7015-6-11 (2008). 18442412 PMC2386807

[b20] Provenzano P. P. . Collagen reorganization at the tumor-stromal interface facilitates local invasion. BMC Med 4, 38, 10.1186/1741-7015-4-38 (2006). 17190588 PMC1781458

[b21] Shields J. D. . Autologous chemotaxis as a mechanism of tumor cell homing to lymphatics via interstitial flow and autocrine CCR7 signaling. Cancer Cell 11, 526–538, 10.1016/j.ccr.2007.04.020 (2007). 17560334

[b22] Pinner S. & Sahai E. Imaging amoeboid cancer cell motility *in vivo*. J Microsc-Oxford 231, 441–445, 10.1111/j.1365-2818.2008.02056.x (2008). 18754999

[b23] Wyckoff J. B. . Direct visualization of macrophage-assisted tumor cell intravasation in mammary tumors. Cancer Res 67, 2649–2656, 10.1158/0008-5472.CAN-06-1823 (2007). 17363585

[b24] Patsialou A. . Invasion of human breast cancer cells *in vivo* requires both paracrine and autocrine loops involving the colony-stimulating factor-1 receptor. Cancer Res 69, 9498–9506, 10.1158/0008-5472.CAN-09-1868 (2009). 19934330 PMC2794986

[b25] Wang S. J., Saadi W., Lin F., Minh-Canh Nguyen C. & Li Jeon N. Differential effects of EGF gradient profiles on MDA-MB-231 breast cancer cell chemotaxis. Exp Cell Res 300, 180–189, 10.1016/j.yexcr.2004.06.030 (2004). 15383325

[b26] Saadi W., Wang S. J., Lin F. & Jeon N. L. A parallel-gradient microfluidic chamber for quantitative analysis of breast cancer cell chemotaxis. Biomed Microdevices 8, 109–118, 10.1007/s10544-006-7706-6 (2006). 16688570

[b27] Yamada K. M. & Cukierman E. Modeling tissue morphogenesis and cancer in 3D. Cell 130, 601–610, 10.1016/j.cell.2007.08.006 (2007). 17719539

[b28] Sung K. E. & Beebe D. J. Microfluidic 3D models of cancer. Adv Drug Deliv Rev 79–80, 68–78, 10.1016/j.addr.2014.07.002 (2014). PMC4258433 25017040

[b29] Zervantonakis I. . Concentration gradients in microfluidic 3D matrix cell culture systems. International Journal of Micro-Nano Scale Transport 1, 27–36, 10.1260/1759-3093.1.1.27 (2010).

[b30] Sung K. E. . Transition to invasion in breast cancer: a microfluidic *in vitro* model enables examination of spatial and temporal effects. Integr Biol (Camb) 3, 439–450, 10.1039/c0ib00063a (2011). 21135965 PMC3094750

[b31] Kim B. J. . Cooperative roles of SDF-1alpha and EGF gradients on tumor cell migration revealed by a robust 3D microfluidic model. PLoS One 8, e68422, 10.1371/journal.pone.0068422 (2013). 23869217 PMC3711811

[b32] Haessler U., Kalinin Y., Swartz M. A. & Wu M. An agarose-based microfluidic platform with a gradient buffer for 3D chemotaxis studies. Biomed Microdevices 11, 827–835, 10.1007/s10544-009-9299-3 (2009). 19343497

[b33] Zervantonakis I. K. . Three-dimensional microfluidic model for tumor cell intravasation and endothelial barrier function. Proc Natl Acad Sci USA 109, 13515–13520, 10.1073/pnas.1210182109 (2012). 22869695 PMC3427099

[b34] Bersini S. . A microfluidic 3D *in vitro* model for specificity of breast cancer metastasis to bone. Biomaterials 35, 2454–2461, 10.1016/j.biomaterials.2013.11.050 (2014). 24388382 PMC3905838

[b35] Jeon J. S. . Human 3D vascularized organotypic microfluidic assays to study breast cancer cell extravasation. Proc Natl Acad Sci USA 112, 214–219, 10.1073/pnas.1417115112 (2015). 25524628 PMC4291627

[b36] Chung S. . Cell migration into scaffolds under co-culture conditions in a microfluidic platform. Lab Chip 9, 269–275, 10.1039/b807585a (2009). 19107284

[b37] Sung K. E. . Understanding the impact of 2D and 3D fibroblast cultures on *in vitro* breast cancer models. PLoS One 8, e76373, 10.1371/journal.pone.0076373 (2013). 24124550 PMC3790689

[b38] Weigelt B., Ghajar C. M. & Bissell M. J. The need for complex 3D culture models to unravel novel pathways and identify accurate biomarkers in breast cancer. Adv Drug Deliv Rev 69–70, 42–51, 10.1016/j.addr.2014.01.001 (2014). PMC4186247 24412474

[b39] Bissell M. J., Radisky D. C., Rizki A., Weaver V. M. & Petersen O. W. The organizing principle: microenvironmental influences in the normal and malignant breast. Differentiation 70, 537–546, 10.1046/j.1432-0436.2002.700907.x (2002). 12492495 PMC2933198

[b40] Nelson C. M. & Bissell M. J. Modeling dynamic reciprocity: engineering three-dimensional culture models of breast architecture, function, and neoplastic transformation. Semin Cancer Biol 15, 342–352, 10.1016/j.semcancer.2005.05.001 (2005). 15963732 PMC2933210

[b41] Huang C. P. . Engineering microscale cellular niches for three-dimensional multicellular co-cultures. Lab Chip 9, 1740–1748, 10.1039/b818401a (2009). 19495458 PMC3758562

[b42] Farahat W. A. . Ensemble analysis of angiogenic growth in three-dimensional microfluidic cell cultures. PLoS One 7, e37333, 10.1371/journal.pone.0037333 (2012). 22662145 PMC3360734

[b43] Ma H., Liu T., Qin J. & Lin B. Characterization of the interaction between fibroblasts and tumor cells on a microfluidic co-culture device. Electrophoresis 31, 1599–1605, 10.1002/elps.200900776 (2010). 20414883

[b44] Wang Q., Villeneuve G. & Wang Z. X. Control of epidermal growth factor receptor endocytosis by receptor dimerization, rather than receptor kinase activation. Embo Rep 6, 942–948, 10.1038/sj.embor.7400491 (2005). 16113650 PMC1369181

[b45] Mutch L. J., Howden J. D., Jenner E. P., Poulter N. S. & Rappoport J. Z. Polarised clathrin-mediated endocytosis of EGFR during chemotactic invasion. Traffic 15, 648–664 (2014). 24921075 10.1111/tra.12165 PMC4309520

[b46] Nikkhah M., Strobl J. S., Peddi B. & Agah M. Cytoskeletal role in differential adhesion patterns of normal fibroblasts and breast cancer cells inside silicon microenvironments. Biomed Microdevices 11, 585–595, 10.1007/s10544-008-9268-2 (2009). 19089620

[b47] Nikkhah M. . MCF10A and MDA-MB-231 human breast basal epithelial cell co-culture in silicon micro-arrays. Biomaterials 32, 7625–7632, 10.1016/j.biomaterials.2011.06.041 (2011). 21764441

[b48] Nikkhah M., Strobl J. S., De Vita R. & Agah M. The cytoskeletal organization of breast carcinoma and fibroblast cells inside three dimensional (3-D) isotropic silicon microstructures. Biomaterials 31, 4552–4561, 10.1016/j.biomaterials.2010.02.034 (2010). 20207413

[b49] Strobl J. S., Nikkhah M. & Agah M. Actions of the anti-cancer drug suberoylanilide hydroxamic acid (SAHA) on human breast cancer cytoarchitecture in silicon microstructures. Biomaterials 31, 7043–7050, 10.1016/j.biomaterials.2010.05.023 (2010). 20579727

[b50] Peela N. . A three dimensional micropatterned tumor model for breast cancer cell migration studies. Biomaterials 81, 72–83, 10.1016/j.biomaterials.2015.11.039 (2015). 26724455

[b51] Even-Ram S. & Yamada K. M. Cell migration in 3D matrix. Curr Opin Cell Biol 17, 524–532, 10.1016/j.ceb.2005.08.015 (2005). 16112853

[b52] Smalley K. S., Lioni M. & Herlyn M. Life isn’t flat: taking cancer biology to the next dimension. In vitro cellular & developmental biology. Animal 42, 242–247, 10.1290/0604027.1 (2006). 17163781

[b53] Mosadegh B., Saadi W., Wang S. J. & Jeon N. L. Epidermal growth factor promotes breast cancer cell chemotaxis in CXCL12 gradients. Biotechnol Bioeng 100, 1205–1213, 10.1002/bit.21851 (2008). 18553401

[b54] Paszek M. J. . Tensional homeostasis and the malignant phenotype. Cancer Cell 8, 241–254, 10.1016/j.ccr.2005.08.010 (2005). 16169468

[b55] Campbell L. L. & Polyak K. Breast tumor heterogeneity: cancer stem cells or clonal evolution? Cell Cycle 6, 2332–2338 (2007). 17786053 10.4161/cc.6.19.4914

[b56] Hughes-Alford S. K. & Lauffenburger D. A. Quantitative analysis of gradient sensing: towards building predictive models of chemotaxis in cancer. Curr Opin Cell Biol 24, 284–291, 10.1016/j.ceb.2012.01.001 (2012). 22284347 PMC3320675

[b57] Ichinose J., Murata M., Yanagida T. & Sako Y. EGF signalling amplification induced by dynamic clustering of EGFR. Biochem Biophys Res Commun 324, 1143–1149, 10.1016/j.bbrc.2004.09.173 (2004). 15485674

[b58] Schulte A. . Glioblastoma Stem-like Cell Lines with Either Maintenance or Loss of High-Level EGFR Amplification, Generated via Modulation of Ligand Concentration. Clinical Cancer Research 18, 1901–1913, 10.1158/1078-0432.Ccr-11-3084 (2012). 22316604

[b59] Friedl P. & Wolf K. Tumour-cell invasion and migration: diversity and escape mechanisms. Nat Rev Cancer 3, 362–374, 10.1038/nrc1075 (2003). 12724734

[b60] Lammermann T. . Rapid leukocyte migration by integrin-independent flowing and squeezing. Nature 453, 51–55, 10.1038/nature06887 (2008). 18451854

[b61] Fraley S. I. . A distinctive role for focal adhesion proteins in three-dimensional cell motility. Nat Cell Biol 12, 598–604, 10.1038/ncb2062 (2010). 20473295 PMC3116660

[b62] Gaggioli C. . Fibroblast-led collective invasion of carcinoma cells with differing roles for RhoGTPases in leading and following cells. Nat Cell Biol 9, 1392–1400, 10.1038/ncb1658 (2007). 18037882

[b63] Kalluri R. & Zeisberg M. Fibroblasts in cancer. Nat Rev Cancer 6, 392–401, 10.1038/nrc1877 (2006). 16572188

[b64] Orimo A. . Stromal fibroblasts present in invasive human breast carcinomas promote tumor growth and angiogenesis through elevated SDF-1/CXCL12 secretion. Cell 121, 335–348, 10.1016/j.cell.2005.02.034 (2005). 15882617

[b65] Loessner D. . Bioengineered 3D platform to explore cell-ECM interactions and drug resistance of epithelial ovarian cancer cells. Biomaterials 31, 8494–8506, 10.1016/j.biomaterials.2010.07.064 (2010). 20709389

[b66] Kothapalli C. R. & Honarmandi P. Theoretical and experimental quantification of the role of diffusive chemogradients on neuritogenesis within three-dimensional collagen scaffolds. Acta Biomater 10, 3664–3674, 10.1016/j.actbio.2014.05.009 (2014). 24830550

[b67] Wang C., Lu H. & Schwartz M. A. A novel *in vitro* flow system for changing flow direction on endothelial cells. J Biomech 45, 1212–1218, 10.1016/j.jbiomech.2012.01.045 (2012). 22386042 PMC3327813

[b68] Stylianopoulos T., Diop-Frimpong B., Munn L. L. & Jain R. K. Diffusion anisotropy in collagen gels and tumors: the effect of fiber network orientation. Biophys J 99, 3119–3128, 10.1016/j.bpj.2010.08.065 (2010). 21081058 PMC2980743

[b69] Sabeh F., Shimizu-Hirota R. & Weiss S. J. Protease-dependent versus -independent cancer cell invasion programs: three-dimensional amoeboid movement revisited. J Cell Biol 185, 11–19, 10.1083/jcb.200807195 (2009). 19332889 PMC2700505

[b70] Guo Y. . Differentiation of clinically benign and malignant breast lesions using diffusion-weighted imaging. Journal of magnetic resonance imaging : JMRI 16, 172–178, 10.1002/jmri.10140 (2002). 12203765

[b71] Lyng H., Haraldseth O. & Rofstad E. K. Measurement of cell density and necrotic fraction in human melanoma xenografts by diffusion weighted magnetic resonance imaging. Magnetic resonance in medicine 43, 828–836 (2000). 10861877 10.1002/1522-2594(200006)43:6<828::aid-mrm8>3.0.co;2-p

