# Supplementary material for: Breast Cancer Cell Invasion into a Three Dimensional Tumor-Stroma Microenvironment
Source: Sci Rep. 2016 Sep 28;6:34094. doi: 10.1038/srep34094 (PMC5039718; doi:10.1038/srep34094)
Supplement: Supplementary Information [file srep34094-s1.pdf]

Breast Cancer Cell Invasion into a Three Dimensional Tumor-Stroma Microenvironment

Danh Truong[1], Julieann Puleo[2], Alison Llave[1], Ghassan Mouneimne[2], Roger D. Kamm[3, 4], Mehdi Nikkhah[1]*

[1]School of Biological and Health Systems Engineering (SBHSE), Arizona State University, Tempe, Arizona 85287, USA.

[2]University of Arizona Cancer Center, Department of Cellular and Molecular Medicine, Tucson, Arizona 85724, USA.

[3]Department of Biological Engineering and [4]Mechanical Engineering, Massachusetts Institute of Technology, Cambridge, MA

*CORRESPONDING AUTHOR:

Mehdi Nikkhah (mnikkhah@asu.edu)

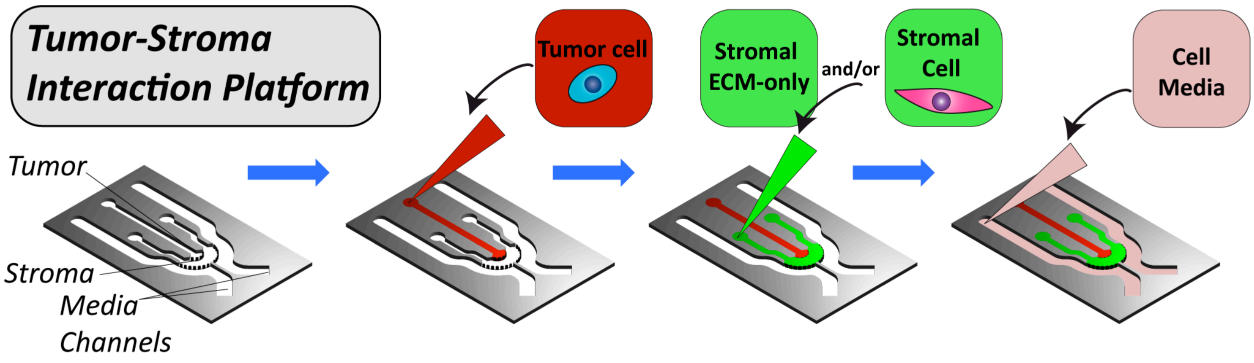

Fig. S1. Detailed schematic of tumor-stroma loading. Cancer cells (red) are injected into the tumor region. The stroma (green), with or without stromal cells, are injected into the stromal region. Media is added into the media channels.

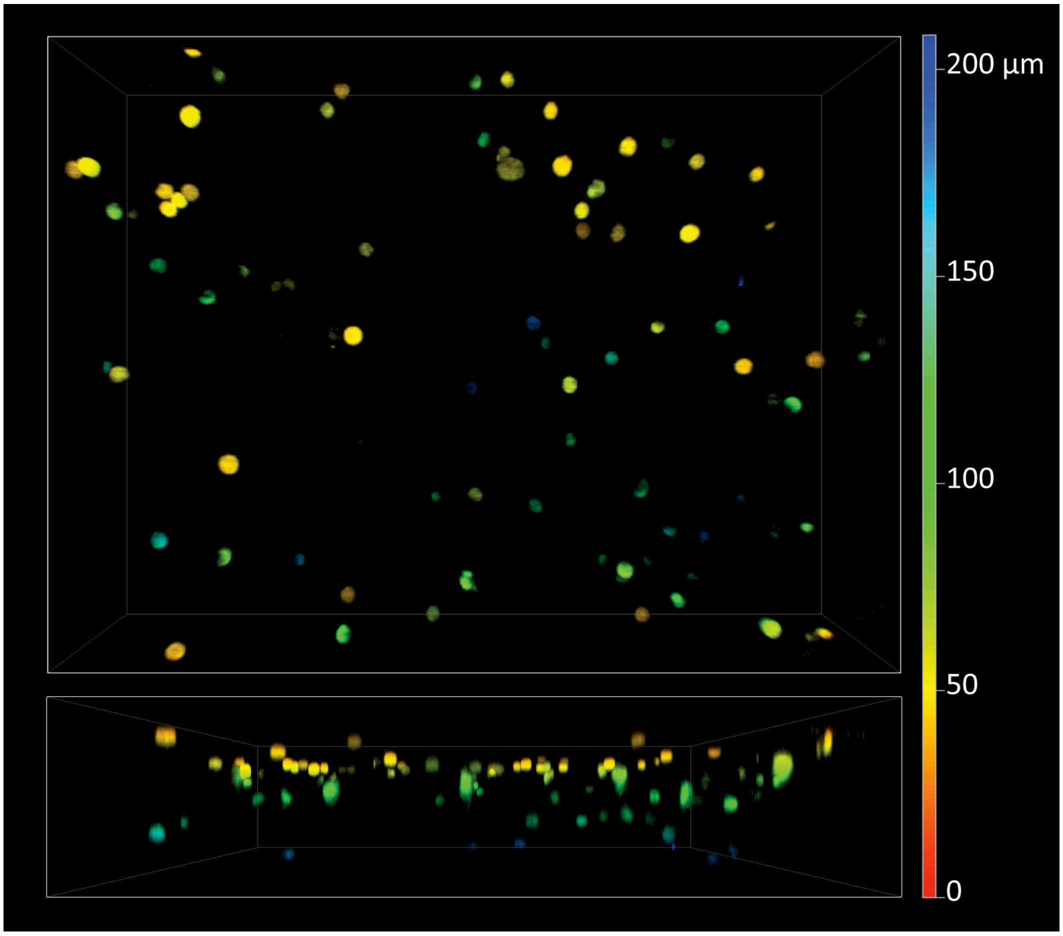

Fig. S2. Depth coding of cells encapsulated within the matrix. The color represents where the cells are located within the 3D space.

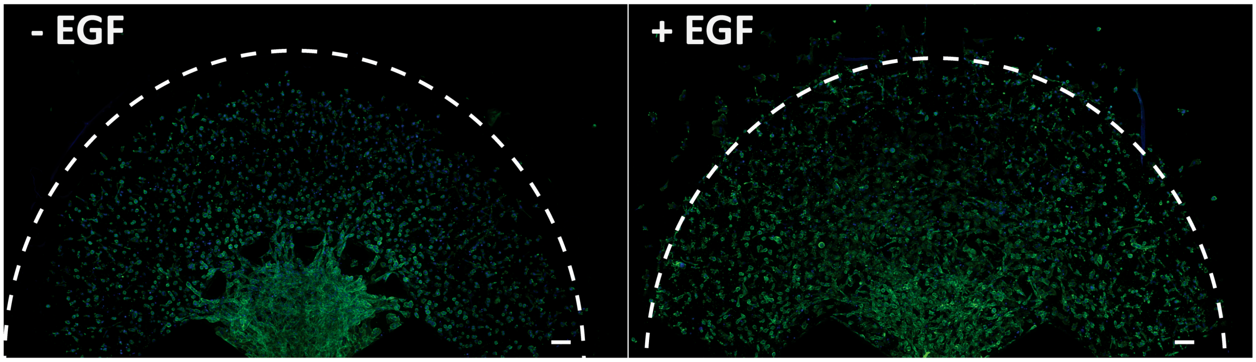

Fig. S3. Whole-device images taken of actin-stained (green) cells in both conditions on day 4. It was apparent that by day 4, more cells within the (+) EGF group reached the area past the stroma (scale bar: 100 μm).

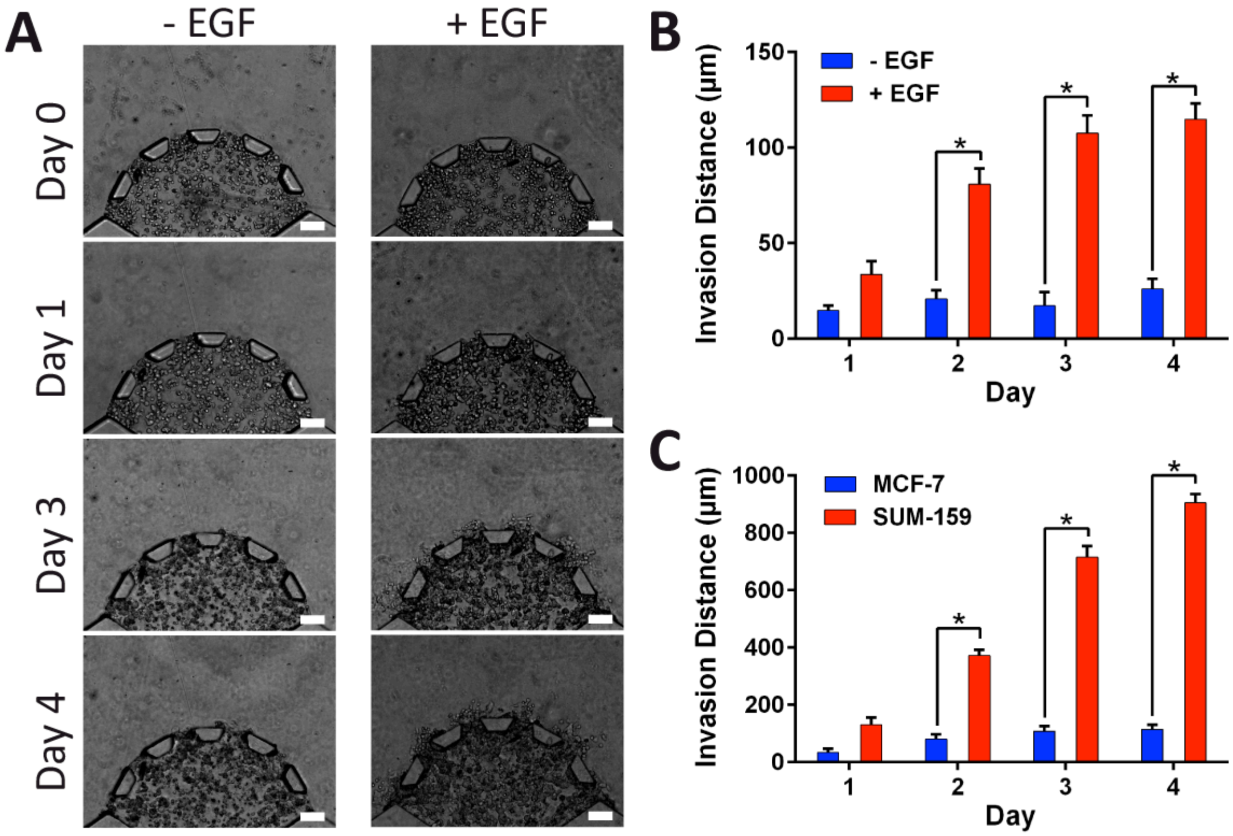

Fig. S4. MCF-7 3D invasion assay. (A) Devices were split into two groups. The (+) EGF group was exposed to 50 ng/mL EGF and the (–) EGF group was not given EGF. (+) EGF MCF-7 cells migrated out from the tumor region by day 2 with slight invasion by day 4. (-) EGF group showed little invasion (scale bar: 100 µm). (B) Invasion distance of the tumor front was calculated from the radial distances of the furthest cells from the tumor region. (+) EGF cells exhibited significantly higher invasion by day 2 ($P < 0.05$ calculated from student's T test with more than three devices for each condition). (D) Comparing (+) EGF groups between SUM-159 and MCF-7 cells showed that SUM-159 had almost 10-fold higher invasion ($P < 0.05$ calculated from student's T test with more than three devices for each condition).

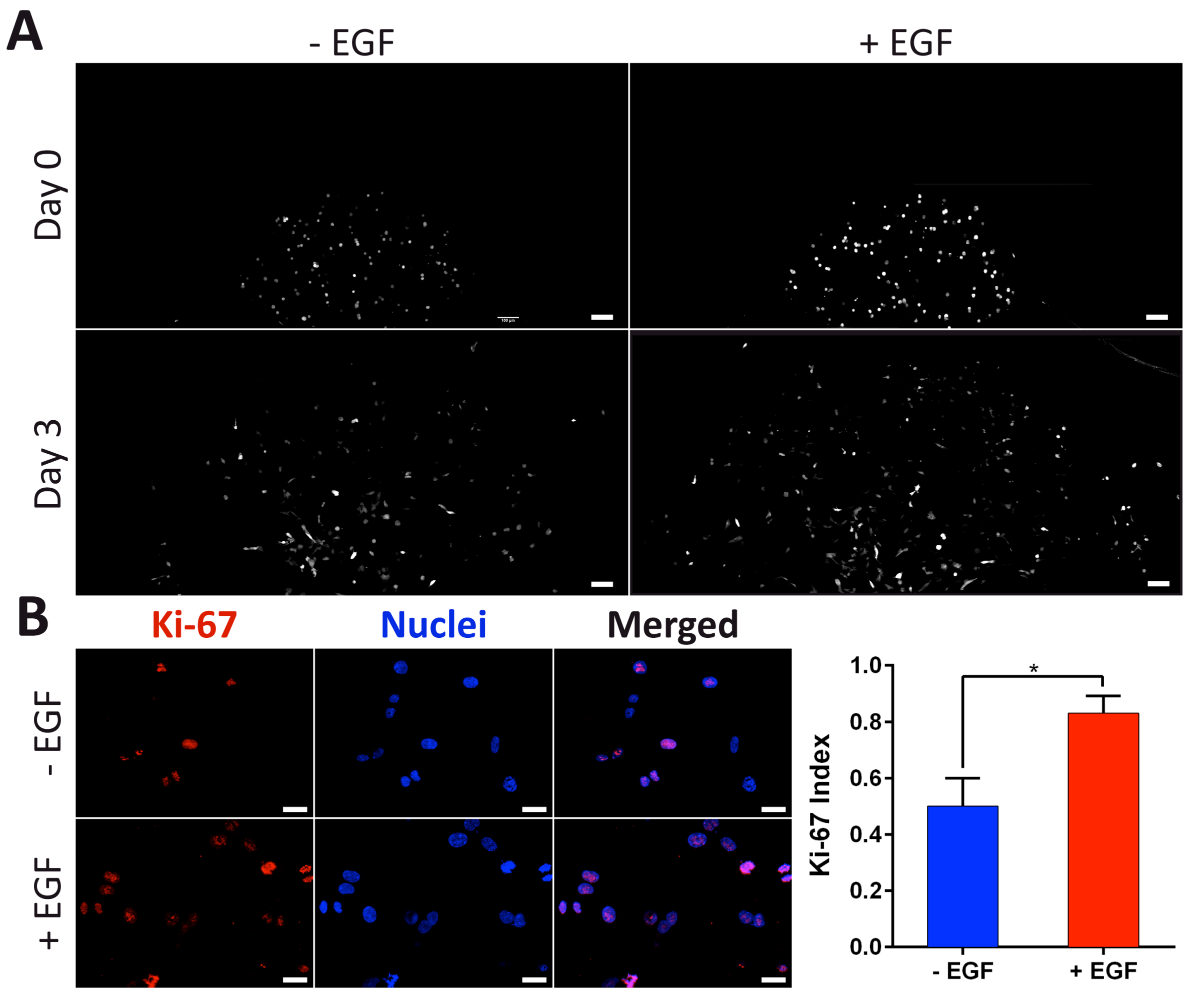

Fig. S5. (A) Cell count was quantified as the increase in fluorescently labeled cells over time (cells were stably expressing mCherry) (scale bar: 100 µm). (B) Ki-67 index was imaged and quantified by comparing expression of Ki-67 (red) and nuclei (blue) (scale bar: 20 µm). (+) EGF group showed significantly higher expression of Ki-67 compared to (-) EGF ($P < 0.05$ calculated from student's T test with more than three devices for each condition).

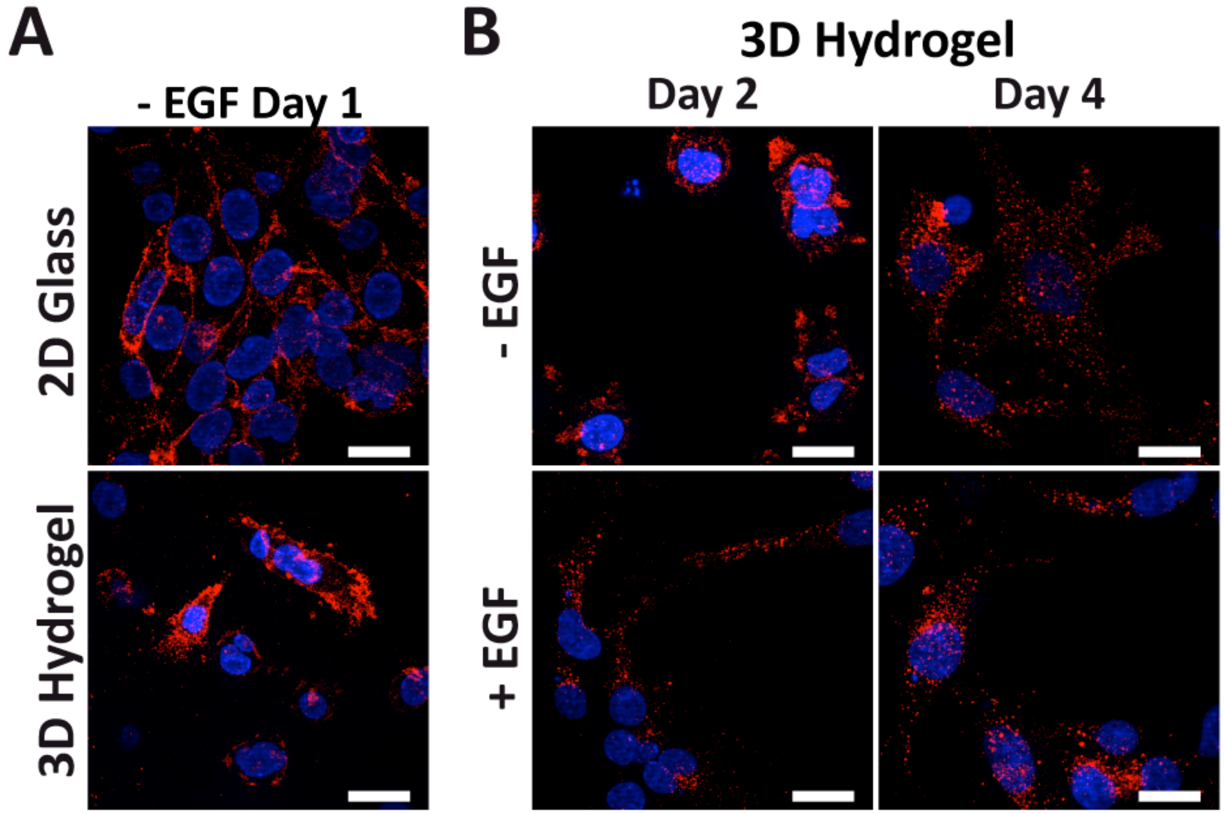

Fig. S6. Investigation of EGFR localization over time. (A) Cells were stained for EGFR (red) and nuclei (blue) (scale bar: 20 μm) within the first 24 h of culture on 2D glass and in 3D in the microfluidic device. Z-projection of cells showed EGFR near the cell periphery. (B) Cells were treated with EGF for 24 h (Day 2) and continuously for 72 h (Day 4). By day 2, punctate EGFR was visible in the cytoplasm of (+) EGF cells while (-) EGF cells maintained EGFR localization at the cell periphery. Day 4 showed that both conditions had punctate EGFRs but (+) EGF displayed clustering toward the nuclei.

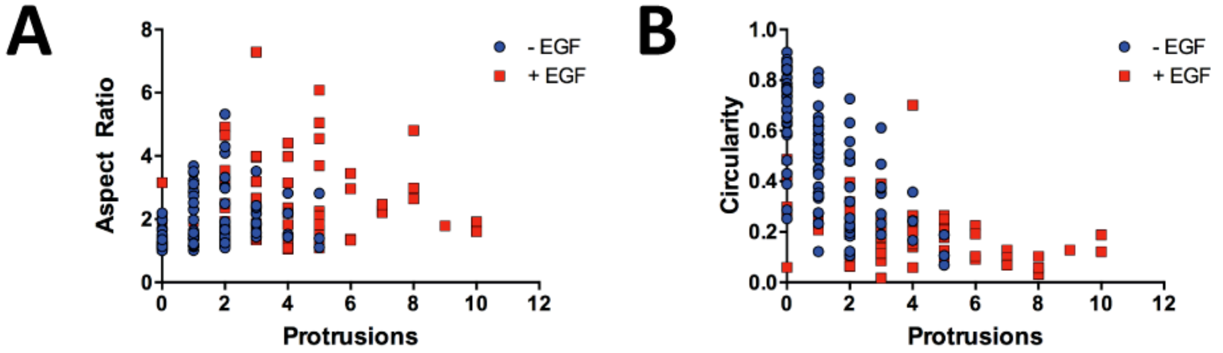

Fig. S7. (A) AR was correlated to protrusions under the two conditions and R-squared for both conditions was determined to be 0.11 ($p < 0.001$) and 0.00 ($p = 0.61$) for (-) EGF and (+) EGF respectively. These results suggested that AR and cell protrusions slightly correlated prior to EGF stimulation, but upon introduction of EGF the correlation was reduced to none or could not be determined. (B) When correlated to circularity, it could be seen under (-) EGF condition that the circularity sharply decreased when cell protrusions increased (R-squared = 0.45, $p < 0.0001$). For (+) EGF, circularity moderately declined with increasing cell extensions where the correlation was weaker than without EGF (R-squared = 0.16, $p < 0.001$).

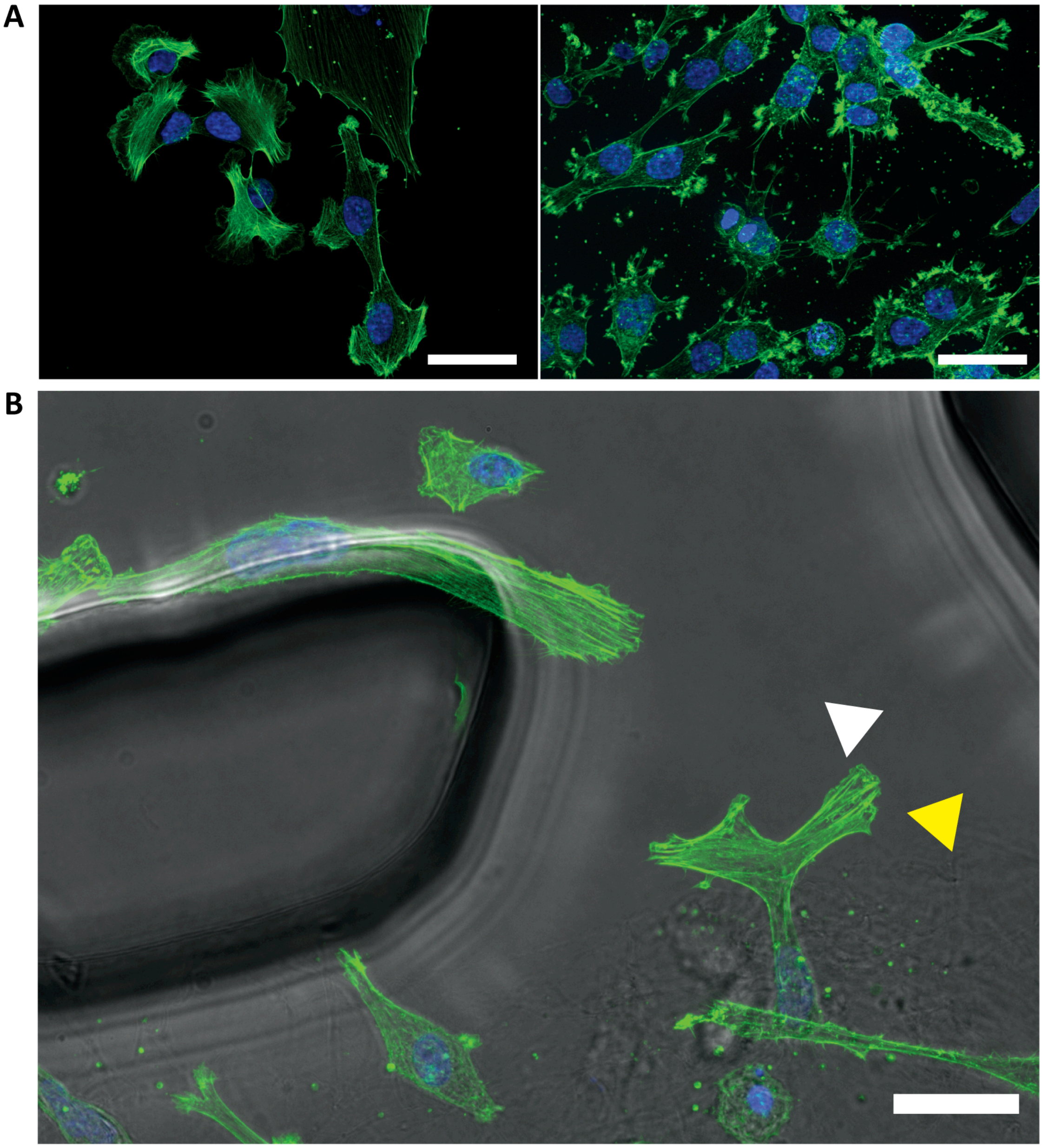

Fig. S8. (A) Z-projection of actin-stained images of cells on 2D substrate (left) and within the 3D matrix (right, scale bar: 25 μm). (B) Z-projection of F-actin staining of cells leaving the collagen matrix (yellow arrow) and protruding toward the glass (white arrow, scale bar: 50 μm).

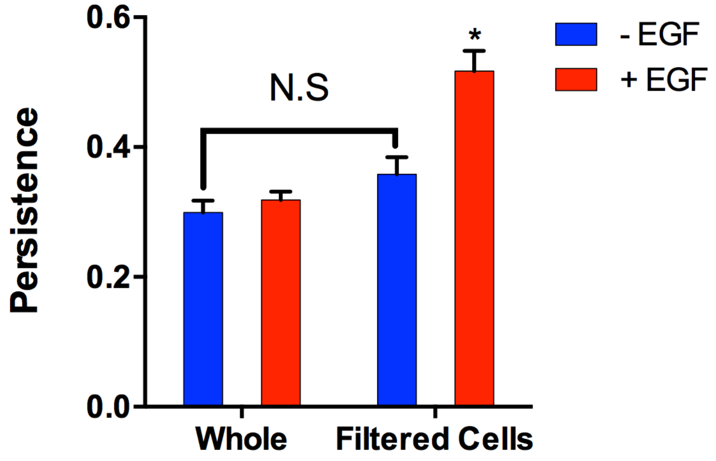

Fig. S9. Analysis of persistence between the whole-population of cells to the filtered cells using a two-way ANOVA and multiple comparison's test. * indicates a significantly different group when $P < 0.05$.

Movie S1. Time-lapse movie of cells migrating throughout the stroma. Movie duration: 18h, time interval: 45 min, scale bar: 200 µm.

Movie S2. Z-projection movie of migrating cells within the stroma matrix.

Movie S3. 3D movie of migrating cells within the stroma matrix (scale bar: 100 µm). The cells were analyzed using the NIS Elements AR Microscope Imaging Software by Nikon. The fluorescent movies were converted to binary and thresholded. The software automatically calculated 3D cell bodies based on the Z-stack images. Next, the cells were tracked using the tracking module, which revealed the differences in cell speed and persistence.

Movie S4. Z-projection of actin (green) and tubulin (red) stained cells in (-) EGF condition.

Movie S5. Z-projection of actin (green) and tubulin (red) stained cells in (+) EGF condition.

Movie S6. Z-projection of actin (green) and tubulin (red) stained cells comparing the cells on 2D substrate and within the 3D stroma.

Movie S7. Z-projection movie of migrating cells on 2D plane.
